# A cluster-randomized controlled trial of the effectiveness of the *JUMP Math* program of math instruction for improving elementary math achievement

**DOI:** 10.1371/journal.pone.0223049

**Published:** 2019-10-30

**Authors:** Tracy Solomon, Annie Dupuis, Arland O’Hara, Min-Na Hockenberry, Jenny Lam, Geraldine Goco, Bruce Ferguson, Rosemary Tannock

**Affiliations:** 1 Department of Psychiatry, Hospital for Sick Children, Toronto, Ontario, Canada; 2 Clinical Research Services, Hospital for Sick Children, Toronto, Ontario, Canada; 3 Dalla Lana School of Public Health, University of Toronto, Toronto, Ontario, Canada; 4 Department of Psychology, University of Toronto, Ontario, Canada; 5 Department of Psychiatry, University of Toronto, Ontario, Canada; 6 Neurosciences and Mental Health, Hospital for Sick Children, Toronto, Ontario, Canada; 7 Applied Psychology and Human Development, Ontario Institute for Studies in Education, University of Toronto, Toronto, Ontario, Canada; French National Center for Scientific Research (CNRS) & University of Lyon, FRANCE

## Abstract

Students in many western countries struggle to achieve acceptable standards in numeracy despite its recognition as an important 21^st^ century skill. As commercial math programs remain a staple of classroom instruction, investigations of their effectiveness are essential to inform decision-making regarding how to invest limited resources while maximizing student gains. We conducted a cluster randomized-controlled trial of the effectiveness of JUMP Math, a distinctive math program whose central tenets are empirically supported, for improving elementary math achievement (clinical trial.gov no. NCT02456181). The study involved 554 grade 2 (primary) and 592 grade 5 (junior) students and 193 teachers in 41 schools, in an urban-rural Canadian school board. Schools were randomly assigned to use either JUMP Math or their business-as-usual, problem-based approach to math instruction. We tracked student progress in math achievement on standardized and curriculum-based measures of computation and problem solving, for 2 consecutive school years. Junior students taught with JUMP Math made significantly greater progress in computation than their non-JUMP peers but the groups did not differ significantly in problem solving. Effects took hold relatively quickly, replicating the results from an earlier pilot study. Primary students in the non-JUMP group made significantly greater gains in problem solving and computation in year 1. But those taught with JUMP Math made significantly greater gains in problem solving and the groups did not differ in computation, in year 2. The positive effects of JUMP Math are noteworthy given that the JUMP Math teachers were likely still adjusting to the new program. That these positive findings were obtained in an effectiveness study (i.e. in real-world conditions), suggests that JUMP Math may be a valuable evidence-based addition to the teacher’s toolbox. Given the importance of numeracy for 21^st^ century functioning, identifying and implementing effective math instruction programs could have far-reaching, positive implications.

## Introduction

Numeracy, the ability to understand and to utilize quantitative information, has been implicated in health management, criminality, financial welfare and employment, with greater numeracy skills related to better outcomes [1–4]. Indeed, employment in STEM fields (Science, Technology, Engineering and Mathematics), which rely heavily on mathematical skills, has been projected to increase three times as much as in non-STEM fields between 2005 and 2024 (33% versus 10%) and STEM graduates experience a higher employment rate and higher earnings than non-STEM graduates [5]. Yet in many western countries students’ mathematical skills remain far from adequate for eventual entry into a globally competitive, STEM-oriented workforce [6–8] (see S1 Text).

Efforts to improve mathematics achievement have focused increasingly on the early school years as early difficulties in mathematics have been linked to later academic and professional success. Individual differences in mathematical skill are already apparent upon Kindergarten entry and predict later academic achievement more strongly than early reading, attention, or socioemotional skills [9]. Persistent difficulty in mathematics is associated with lower rates of high school graduation and college entry and high school mathematics achievement predicts college graduation, career earnings and earnings growth [10, 11].

Much of the discussion has centered on the curriculum—what children should be taught, when and how they should be taught it (see for example, [12]). Curriculum expectations by grade are established by policy makers and typically delivered to students by way of commercial, textbook based programs. Indeed, a survey of almost 6000 math and science teachers indicated that 85% of elementary classrooms in the United States rely on at least one such program [13]. Yet studies investigating the effect of math programs on math achievement are a relatively recent phenomenon. Moreover, many studies lack scientific rigour and those that meet criteria for rigour often yield limited, and sometimes mixed, evidence regarding program impact (see [14–17]). Thus, we are only beginning to understand the extent to which math instruction programs influence math learning.

The limited evidence regarding the impact of math programs on math learning might lead Education stakeholders to focus on factors other than instruction to improve math achievement (e.g. class size, teacher preparation), while continuing to invest in programs whose impact remains unknown. Randomized-controlled trials (RCT’s) of math instruction programs are essential to determine their contribution to math achievement and to identify the programs that are most effective for improving it.

### Program descriptions

We investigated the effectiveness of JUMP Math (JUMP), a distinctive approach to math instruction, developed by a Canadian mathematician based on a wealth of experience working with children with diverse math skills and challenges [18, 19]. The program is fully developed for K-8 and currently reaches more than 210, 000 students worldwide, with rapidly growing interest in Canada and the United States. It has been adapted to meet regional curriculum expectations (e.g. there is an American common core version), translated into multiple languages including French, Spanish and Bulgarian (translation into Inuktitut, a Canadian indigenous language, is also underway), and aims to be affordable (JUMP Math is a registered charity). Informal study in diverse settings suggested the potential for a positive impact on math learning [19]. JUMP instruction includes problem solving but the program is distinct from problem-based learning, the prevailing approach to math instruction in a number of countries, and its key ideas are empirically supported.

To elucidate the nature of JUMP instruction, it is instructive first to consider the central features of problem-based learning. Table 1 provides a summary of the key contrasts between the two curricula.

**Table 1:** Summary of key contrasts between problem-based math and JUMP math.

	Program Feature	Problem-Based Math^a^	JUMP Math^b^
1.	Teacher’s Role	Facilitate the discovery process, little to no direct instruction	Provide direct instruction to guide discovery of target ideas
2.	Knowledge Construction	Through physical and mental activity	Limited physical activity, more emphasis on mental activity
3.	Manipulatives	Freely available, exploratory use to aid discovery of concepts required to solve word problems	Limited, prescribed use to discover target concepts, removed as soon as concepts grasped, more emphasis on symbolic math
4.	Algorithms and Practice	Less emphasis on algorithms, practice, and fact fluency, more on conceptual learning to avoid memorization without understanding	Algorithms taught along with conceptual understanding, practice considered essential for proficiency/acquiring fact fluency
5.	Problem-Solving	Lessons center on real-world problems with multiple possible solutions	Balance of abstract and real-world problems that increase in complexity with the development of math and language skills
6.	Technology	Calculators permitted even in early years to free up cognitive resources for the discovery process	Calculators eschewed in favour of mental math, distinctive finger counting method taught as part of program
7.	Language Demands	Relatively high; students read complex word problems and explanatory text and justify their work orally, in writing (using graphics, words and symbols)	Relatively low; word problems pared down to essentials, become more linguistically complex over time, explanatory text reserved for teacher’s guides
8.	Assessment	Summative and cumulative	Frequent/ongoing, to ensure understanding before introducing new material
9.	Configuration of Students in Classroom	Mostly collaborative, small-group work, creative use of classroom space, whole class gathers for problem presentation and to share strategies, solutions	Mostly individual, desk work, occasional work with a partner, whole class gathers for direct instruction and sharing of strategies, solutions

^a^see [21–23]

^b^see [18,19]

Problem-based learning (see note [20]), [21–23] aims to develop the critical and creative mathematical reasoning children will eventually require in the modern workplace. A key feature is to make mathematics meaningful to children, to engage their attention and to promote a deep conceptual understanding of the material. Students are presented with real world problems and typically work collaboratively to determine, and then solve, the mathematics involved. Teachers facilitate learning, rather than instruct students directly. Children are encouraged to manipulate concrete materials (a wide variety of objects, pictures and so on) that are freely available, to discover target ideas. Calculators are permitted as early as Kindergarten to alleviate computational demands, thus leaving working memory resources free for the discovery process. Problem-based programs vary in their view of practice but typically eschew algorithms to encourage children to look beyond mathematical procedures and fact memorization and to focus on reasoning about the underlying concepts. Language plays a central role. With the emphasis on contextually rich problems and the inclusion of considerable explanatory material, mathematics texts tend to be linguistically dense and even very young students are required to communicate their thinking orally and in writing (using graphics, symbols and words). Indeed, explaining how they arrived at a problem solution is an essential part of problem-based lessons and considered a key indicator of children’s understanding of the material. For example, a grade 3 word problem retrieved from a well-known, online elementary teacher resource serving teachers in the region where the present research took place reads: “There are 30 legs in my backyard, but I'm counting dogs and kids. How many dogs and kids are in my backyard? Show different solutions to this problem”. Children are provided with manipulatives to help solve the problem and asked to show their solutions using “pictures, numbers and words”. [24] Assessment is both summative and cumulative, although supplementary diagnostic tools are increasingly called upon to help track progress.

In a typical problem-based lesson, the teacher presents a real-world word problem that is open-ended (has multiple possible solutions). Students then work collaboratively, in small groups, to solve the problem using a variety of materials and recording their work using graphics, symbols and text. Teachers circulate to check on progress, facilitate thinking and provide encouragement. The class then reconvenes and the teacher facilitates discussion as the groups share their problem-solving strategies and solutions.

In contrast, in the JUMP program concepts are reduced to increasingly smaller steps until students become proficient at executing that step, then built back up incrementally to meet the curriculum demands. A hallmark of the program is that lessons are finely scaffolded (guided) to help students arrive at target concepts–a process known as “guided discovery”. Moreover, the amount of scaffolding is tailored according to individual needs, allowing the whole class to work on the same concept simultaneously, while individual students vary widely in terms of where they are in the learning process. For example, in a lesson on perimeter in which irregular, right angled shapes are presented with some side lengths given and children must determine the length of the remaining sides, struggling students may work on relatively simple shapes with only one side length missing and lengths in the single digits, while more proficient students may receive problems with more complex shapes, multiple missing side lengths or lengths in the hundreds or thousands. The idea is for children at all levels of functioning to experience success at doing math and to associate this success with their efforts (see S1 Text).

A variety of models (concrete materials, diagrams, charts) are used purposefully in lessons explicitly linking the materials to mathematical ideas such as connecting 3 blocks to the magnitude “3”. However, the idea is to move students’ thinking from the concrete models towards abstract symbols (“3”, “x”) as soon as they have grasped the underlying concepts. For example, in an early algebra lesson in which blocks and a paper bag are used to represent quantities and a missing value, the blocks are gradually replaced with drawings of squares and then Arabic numbers, and the bag with empty squares, short underscores and then “x”, as the teacher works through the lesson. Paper and pencil work is favoured over calculators although students are taught and encouraged to use a unique finger counting method to aid mental computation. (see S1 Text). Moreover, substantial attention is directed to helping students acquire mathematical fact fluency.

JUMP aims to limit working memory and language demands. Much of the explanatory material is reserved for the teacher’s guides so that teachers can control the timing and the amount of information the children receive. In lieu of textbooks, students receive extensive practice working with the lesson ideas in practice and assessment books that contain worked examples and include real world problems pared down to the essential text. For example, at the end of a lesson on sums and differences younger children may be asked to solve problems such as “Midori had 35 pencil crayons. She lost 4. How many does she have left?” Problem complexity increases over time, as children are able to cope with greater mathematical and linguistic demands. Assessment at every step of the learning process is considered critical, to ensure understanding before moving on to new material. This may be as simple as asking “yes/no” questions, asking children to solve a specific problem or set of problems, to demonstrate their developing knowledge of the target concept. Clarifications are given as needed to help students understand why problem solutions work.

A typical JUMP lesson involves multiple iterations of relatively short periods of finely-scaffolded, direct instruction—explicit teaching of concepts and procedures, including the use of worked examples—given to the whole class, followed by plenty of individual practice (and occasional work with a partner) working with the lesson ideas (for a detailed description of a JUMP Math lesson see S1 Text). Teachers circulate to check on progress, provide feedback and encouragement. Example questions are taken up with the whole class with clarification as needed to share strategies, fill in knowledge gaps, and to verify comprehension before introducing new material.

### Empirical support

The central tenets of JUMP math are well supported by the empirical evidence. Indeed, a meta-analysis comparing the effectiveness of unassisted discovery, explicit instruction and enhanced discovery found that explicit instruction was more effective than unassisted discovery, and that enhanced discovery incorporating scaffolding, worked examples, elicited explanations and feedback (all key features of JUMP) was the most beneficial for learning. [25] Another meta-analysis of five decades of research provides evidence for the positive effects of direct instruction on learning. [26]. Numerous studies have shown that children who attribute their success to their efforts are more likely to persevere in the face of challenges than children who attribute their success to inherent ability, ultimately leading to greater success. [27] Gradually fading out the concrete properties of manipulatives and moving towards abstract symbols has been shown to be more effective for learning than moving directly from concrete manipulatives to symbols [28, 29]. Indeed there is evidence that generic symbols (such as υ) may be more effective than concrete instantiations (such as detailed renderings of actual objects) for learning and transfer. [30]. There is also evidence of greater benefits from generating answers to calculation problems, compared to using a calculator, [31] and that finger counting can be an effective aid, especially for novice or struggling mathematicians. [32] Reducing problems to smaller, more manageable components is known to help alleviate working memory demands [33] and there is abundant evidence of the benefits of practice for skill acquisition. [34] The finding that children who have difficulty in math only fare better in math than children who struggle in both reading and math suggests that limiting the language demands of math materials may facilitate math processing. [35] Paring the student materials to only the essential information and allowing teachers to determine how much information to share, and when to share it with students thus takes children’s language and working memory limitations into account [36]. Finally, the development of professional tools to help track student progress (see e.g. [37]) as well as the emphasis on ongoing assessment in the current Response to Intervention model [38], attest to the importance of frequent assessment for effective instruction. Taken together, these findings suggest that the central principles of the JUMP program are well rooted in the scientific literature.

### Present research

We first report a pilot study followed by a larger scale RCT investigating the impact of the JUMP program on elementary math achievement. In both studies, we were interested in the program’s effectiveness (impact in typical, real-world conditions), as opposed to its efficacy (potential impact in ideal conditions) for improving student math achievement. The pilot study aimed to determine if an RCT was feasible and warranted. Feasibility was important because there is no precedent for RCT’s in early Education in Canada. The pilot study focused on grade 5 (as the JUMP materials for grades 5/6 were the most refined at the time) and assessed the students’ progress over 5 months of instruction, largely in computation, given the program’s greater attention to mental math and practice. We reasoned that if JUMP was at all effective, we ought to see an impact here. The larger-scale RCT aimed to replicate and extend the pilot findings. The study involved teachers and students in grade 2 (7 year-olds) and grade 5 (10 year-olds) in a different school board. We evaluated teachers’ implementation fidelity and tracked student progress over two consecutive school years (through the end of grades 3 and 6) on a broad range of math outcomes that included students’ ability to apply their mathematical knowledge, widely regarded in contemporary education as a key indicator of math understanding. [21, 22]

In the scale-up RCT, we investigated the impact on math achievement in year 1, the summer, and year 2 of the study, to shed light on the trajectory of any effects of curricula. We included the summer period because math achievement is particularly vulnerable to so called “summer slide”, resulting in time spent at the beginning of the new school year reviewing the previous year’s work that could otherwise be directed towards learning new material [39]. Smaller summer losses would also indicate that more of the previous year’s material has been successfully transferred to long-term memory, which is an important indicator of learning [40]. Curricula that minimize summer losses could thus help students make greater advances during the school year. Given their extensive experience with problem solving, we expected that students who received their usual methods of math instruction would have an advantage on the problem solving measures, at least in the first year when both students and teachers in the JUMP group were adjusting to the new program. Given the greater attention given to mental math and practice in the JUMP program compared to problem-based learning, we anticipated that any positive effects of JUMP might first be discernible on the computational measures. At issue was the longer- term impact of the two curricula on the various math outcomes.

## Pilot study

### Overview

The pilot study involved grade 5 teachers and students in a rural school board (district; SB1) in Ontario, Canada. Participating schools were randomly assigned to use either JUMP Math (JUMP) or their business-as-usual approach to math instruction (SB1) for about 5 months, including mandatory school holiday breaks of about 3 weeks. We tracked student progress in math achievement, collected demographic data from the teachers and solicited feedback regarding their experience working with their assigned curricula (see S1 Text).

### Ethics statement

The Research Ethics Boards (REB) at the Hospital for Sick Children, Toronto, Ontario, Canada and the participating school board approved the study procedures. Participation was voluntary. We obtained written, informed consent from parents and teachers prior to random assignment to curricula and assent from the children prior to testing. Other than the JUMP Math materials distributed to teachers in the JUMP group (at no cost), teachers did not receive any compensation for participating.

### Materials and methods

#### Participants

Twenty-one principals consented to participate. Schools were randomly assigned to either the JUMP group (11 schools) or to the SB1 group (10 schools). However, 2 schools in the SB1 group declined after random assignment, which together with variation in the size of the participating schools, resulted in an imbalanced number of participating teachers and students in the two groups. The elementary student population in the participating school board was predominantly white, with middle to lower middle class backgrounds and low average, to average academic achievement (see Table 2 and S1 Text).

**Table 2:** Summary of student characteristics at baseline.

	Pilot Study		Scale-Up RCT			
	Jump Math	School Board 1	JUMP Math		School Board 2	
	Grade 5 Mean (sd)	Grade 5 Mean (sd)	Grade 2 Mean (sd)	Grade 5 Mean (sd)	Grade 2 Mean (sd)	Grade 5 Mean (sd)
Number of teachers (T) and students (S) *at baseline*	n = 18T n = 163S	n = 11T n = 107S	n = 31T n = 350S	n = 27T n = 348S	n = 26T n = 204S	n = 22T n = 244S
Student Age in Years	10.4 (0.3)	10.5 (0.3)	7.2 (0.3)	10.2 (0.3)	7.2 (0.3)	10.2 (0.3)
Days Absent (in Yr1 for scale-up)	8.3 (7.2)	8.2 (6.1)	8.4 (6.9)	9.2 (7.6)	10.5 (11.6)	12.0 (16.4)
Days Absent Yr2	-	-	9.0 (8.1)	11.0 (9.5)	8.1 (7.1)	9.9 (9.0)
Hours of Math Instruction/Week (in Yr1 for scale-up)	3.8 (1.0) range: 2.0–6.0	4.0 (0.8) range: 3.0–5.0	5.0 (1.0) range: 2.0–8.3	5.2 (1.4) range: 3.0–8.3	5.1 (1.1) range: 2.7–7.0	5.0 (0.9) range: 3.3–9.3
Hours of Math Instruction/Week Yr2	-	-	4.9 (1.0) range: 3.3–8.8	4.9 (0.7) range: 3.7–6.7	5.4 (1.0) range: 4.2–8.3	5.4 (1.1) range: 3.8–8.3
**Math Achievement**						
Broad Math Cluster	-	-	99.2 (14.2)	88.9 (12.2)	100.0 (16.3)	89.8 (13.1)
Math Fluency	87.0 (10.7)	86.4 (11.3)	91.4 (13.4)	87.2 (12.5)	92.8 (13.6)	87.4 (13.9)
Calculation	86.2 (12.2)	86.6 (13.4)	97.9 (13.9)	83.6 (11.7)	100.3 (14.9)	84.2 (14.2)
Quantitative Concepts	99.7 (13.7)	98.8 (14.8)	-	-	-	-
Applied Problems	-	-	102.2 (13.7)	96.3 (11.9)	101.0 (15.3)	97.0 (11.5)
Problem-Solving Process (PSP)	-	-	18.9 (8.2)	13.0 (5.8)	18.2 (7.9)	13.7 (6.7)
Curriculum Based Computation (CBC)	-	-	16.6 (9.6)	17 (10.5)	15.9 (9.7)	15.5 (9.9)
**Additional Variables**						
Broad Reading Cluster	-	-	102.5 (13.2)	93.6 (13.6)	100.5 (15.0)	94.5 (13.3)
Letter-Word Identification	99.6 (12.5)	99.3 (12.2)	-	-	-	-
Verbal IQ	99.6 (10.5)	97.8 (11.9)	103.0 (11.3)	96.4 (11.5)	103.0 (11.6)	96.2 (13.3)
Non-Verbal IQ	99.8 (15.5)	102.4 (13.5)	95.8 (15.4)	94.0 (16.4)	97.2 (15.2)	96.8 (14.9)
Working Memory	3.6 (1.3)	3.9 (1.4)	2.7 (1.0)	3.5 (1.3)	2.6 (1.1)	3.4 (1.2)
Processing Speed-Numbers	-	-	101.4 (14.0)	100.5 (11.9)	101.7 (12.8)	102.5 (12.3)
Processing Speed-Letters	-	-	102.8 (12.3)	98.0 (11.6)	101.8 (12.3)	99.3 (11.6)

Teachers were eligible to participate if they were accredited to teach in Ontario, in good standing, and did not plan to take a leave of absence during the study period. Eighteen JUMP and 11 SB1 teachers in the participating schools agreed to take part. As a group, the SB1 teachers had somewhat more teaching experience and somewhat stronger math backgrounds. (see S1 Text).

Students were eligible to participate if they had sufficient grasp of English and did not have developmental challenges serious enough to preclude full participation in the curriculum, as judged by their teacher. Eight to 10 students were randomly selected from each class for tracking, yielding a total of 270 students; 163 JUMP and 107 SB1. Boys and girls were roughly equally represented. Attrition was low; 7 additional students tested at baseline (about half in each group) moved to a non-study school prior to post data collection. The left side of Table 2 shows a summary of the pilot study student characteristics at baseline, which were highly similar in the two groups (see note [41]), [42–44].

#### Teacher professional development

Teachers in both groups received 2 full days (1 in the fall, 1 mid-year) of professional development (PD), according to their assigned curriculum (JUMP or SB1). The content and delivery of the PD was determined by the JUMP organization and by the participating school board, respectively. Members of our research team attended all of the PD sessions to ensure alignment with assigned curricula. We asked teachers to use only their assigned curriculum for the study duration.

The SB1-PD was in line with the business-as-usual, problem-based approach to math instruction and was delivered by members of the board pedagogical support team. The sessions comprised a review of regional expectations regarding the content and pedagogical approach for elementary math instruction, suggestions for implementation, and pedagogical demonstrations including small group work on problem solving. Teachers were given opportunities for questions and discussion. Their key resources following the PD included approved commercial textbooks, the accompanying teacher’s guides and student workbooks normally provided by the school board. As per usual practice, teachers were free to supplement with any other additional materials according to their student’s needs and continued to receive input from the board’s teaching support team, as needed.

Teachers in the JUMP group received the same PD that could be provided to any teacher adopting the program, which was delivered by a professional from the JUMP Math organization. The sessions consisted of numerous pedagogical demonstrations to introduce key program principles, guidance on how to use the teacher’s guides and student practice and assessment books, and suggestions for how to adapt the program for struggling and also for more advanced students. Teachers were given opportunities to ask questions and participate in discussion. Their key resources following the PD were grade specific teacher’s guides containing detailed, explicit lessons for the entire school year with suggestions for how to adapt the materials according to student’s math skills, and the student practice and assessment books containing a wealth of practice material (there are no student text books). Teachers were also encouraged to submit queries for input from the JUMP organization, as needed.

#### Measures and procedures

The math achievement outcome measures included the math fluency, calculation and quantitative concepts scales from the Woodcock-Johnson Achievement Battery (WJ-III [45]). We measured reading achievement with the letter word identification test also from the WJ-III, IQ with the Kaufman Brief Intelligence Test (KBIT; [46]), and verbal working memory with the backwards version of the non-word letter span test adapted from the Wechsler Intelligence Scale for Children (WISC-III [47]). We assessed math and reading achievement at baseline and post-intervention, and IQ and working memory at baseline only (see Table C in S1 Text).

Teachers completed a questionnaire probing their teaching experience, highest level of math education and the amount of math instruction time per week provided to their participating class. They also rated their math teaching experience during the study period on a 10-point scale from “very poor” (0) to “excellent” (10). Teachers in the JUMP group indicated if they would use their assigned curriculum again and if they would recommend it to other teachers.

Students were tested individually in a quiet area in their school by a local team of retired teachers who were familiar with the school board but blind to the study hypothesis and to random assignment to curricula. The study measures were administered in two sessions that took place on separate days. Breaks were given as needed.

### Results

**Fig 1:**
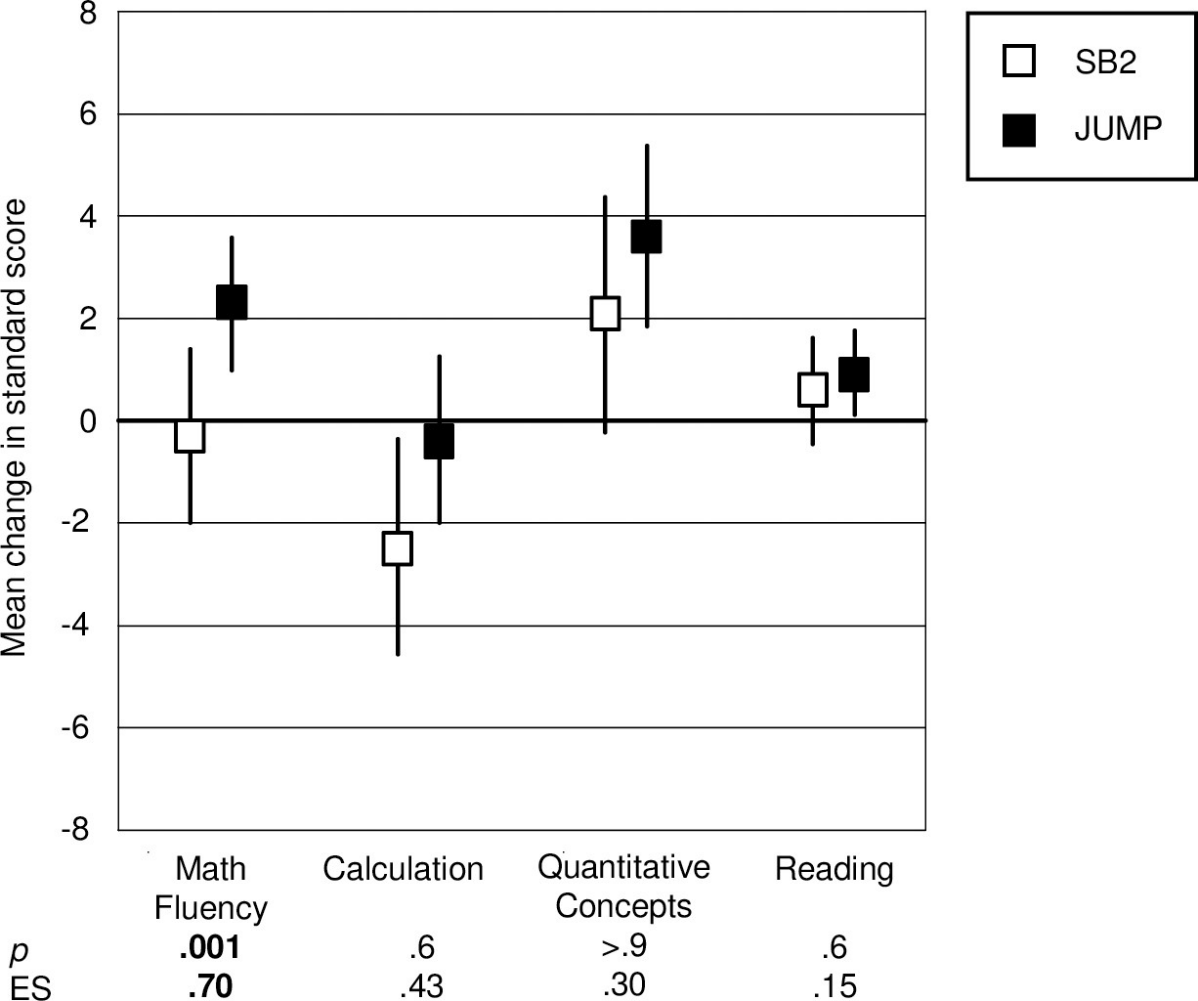
Pilot study results. Results are based on standard scores and therefore indicate progress relative to same aged peers, which is represented by the 0 line. Vertical lines indicate 95% confidence limits around mean change scores. *P*-values and effect sizes (ES) are for the difference between the group means. Vertical lines that *do not* intersect the zero line indicate mean change that is significantly different from expected change, based on available norms.

We used PROC MIXED in SAS 9.3 (copyright 2002–2012 SAS Institute Inc., Cary, NC, USA) to model the change in each outcome as a repeated measures hierarchical regression model. We entered baseline scores on the outcome variable, curriculum (JUMP, SB1), reading achievement at baseline, and gender. The results for the pilot study are shown in Fig 1. (see also Table D in S1 Text).

Note that effect sizes (ES) were derived from estimates from the repeated measures hierarchical regression models. We calculated standard deviation for the pre-post change by multiplying the standard error of the estimate of change for each curriculum by the square root of its degrees of freedom. Effect size is the change in the JUMP group minus the change in the SB1 group, divided by the pooled standard deviation. Hence, a positive value indicates better performance in the JUMP group, a negative value better performance in the SB2 group. An effect size of .25 is considered educationally meaningful [48]. A recent review of educational intervention research found that effect sizes are rarely as large as .30. Hence, values of .50 would be considered very large indeed [49].

As the figure shows, students who received JUMP instruction generally progressed more than their SB1 peers on all of the math achievement measures. The impact was strongest for math fluency, where the group difference was significant (mean change scores -.3 SB1, 2.3 JUMP, *p* = .001, ES = .7 math fluency; -2.5 SB1, -0.4 JUMP, *p* = .6, ES = .43 calculation; 2.1 SB1, 3.6 JUMP, *p* >.9, ES = .3 quantitative concepts). Moreover, comparing performance to expected change based on available norms we found that in the JUMP group, progress was significantly greater than expected on math fluency (*p* = 0.001) and quantitative concepts (*p* = .0003), and not significantly different from expected progress on calculation (*p* = .63). In the SB1 group, progress was not significantly different from expected on math fluency (*p* = .72) or quantitative concepts (*p* = .07), and significantly less than expected on calculation (*p* = .02). The difference between groups was not significant and relatively small for reading (mean change 0.6 SB1, 0.9 JUMP, *p* = .6, ES = .15) compared to the differences on the math measures, although only the JUMP group made significantly more than expected gains here, based on available norms (*p* = .27 SB1; *p* = .03 JUMP) (see S1 Text).

Teachers in the JUMP group rated their experience teaching math during the study period significantly more positively than teachers in the SB1 group (mean ratings 7.2 SB1, 8.2 JUMP, *p* = .03). All of the teachers who used JUMP indicated that they would use it again and that they would recommend it to other teachers.

### Discussion

The pilot study results provided preliminary evidence of a positive impact of JUMP on grade 5 students’ math progress after just a few months of instruction. The impact was strongest on math fluency, but the JUMP group generally made more progress than their school board peers (compared to expected growth based on test norms). These findings are noteworthy given the relatively short study duration and our use of standardized measures, which can be insensitive to small but important changes especially in the short-term. JUMP instruction had relatively little impact on reading suggesting the program effect was specific to math progress, as opposed to more general pedagogical gains. Attrition was low and teacher feedback regarding working with the program was positive. As such, the pilot study results suggested that a scale-up RCT was both feasible and warranted.

## Scale-up RCT

### Overview

The scale up study was a registered cluster-randomized controlled trial—clinical trials.gov identifier NCT02456181 –conducted in a different Ontario school board (district; SB2) serving a small city and the surrounding rural counties. The student population was therefore an urban/rural mix. The study involved 41 schools, 193 teachers and 1146 students.

Table 3 provides an overview of the study design. Schools were randomly assigned to use either JUMP Math (JUMP) or the school board’s business-as-usual problem-solving approach (SB2) to teach math for two consecutive school years. The students were in grade 2 or grade 5 at the start of the study and we tracked their progress in math through the end of grades 3 and 6. Hence, the study included a primary division (grades 2/3) and a junior division (grades 5/6) elementary school cohort (in Canadian elementary schools the primary and junior divisions refer to grades K-3 and 4–6, respectively). The teachers who participated in year 1 were those who taught the study students in grades 2 or 5. The teachers who participated in year 2 were those who inherited and taught the study students in grades 3 or 6.

**Table 3:** Overview of the scale-Up RCT.

	Year 1			Year 2		
Curriculum	Grade	Data Collection	Amount of JUMP Instruction at Year End	Grade	Data Collection	Amount of JUMP Instruction at Year End
JUMP	2	Fall, Spring	1 year	3	Fall, Spring	2 years
	5	Fall, Spring	1 year	6	Fall, Spring	2 years
SB2	2	Fall, Spring	─	3	Fall, Spring	─
	5	Fall, Spring	─	6	Fall, Spring	─

SB2 denotes school board 2, the group that received the business-as-usual, problem based math instruction.

Teachers received PD in their respective curricula, which we asked them to use for the duration of the school year. We assessed student’s math achievement at 4 time points—the fall and spring of each of the two school years (T1and T2 for year 1, and T3 and T4 for year 2). This allowed us to examine change in achievement in 3 time periods; year 1 (T1 to T2), the summer (T2 to T3) and year 2 (T3 to T4). We also assessed teachers’ fidelity of implementation of both types of instruction, around the middle of the each of two school years, by videotaping them as they taught math to their participating classes. As in the pilot study, we tracked student progress in reading as well as math to determine if any effects of JUMP were specific to math learning. To help characterize the groups at baseline we also assessed students’ IQ, working memory and processing speed. Finally, we collected demographic data from the teachers and solicited feedback regarding their experience working with their assigned curricula.

### Ethics statement

The Research Ethics Board (REB) at the Hospital for Sick Children, Toronto, Ontario, Canada and the participating school board approved the study procedures. Participation was voluntary. We obtained written informed consent from teachers; in year 1, prior to randomization to curricula and in year 2, after the curricula were already in place. We obtained separate written, informed consent from teachers for the videotaping. We obtained written, informed consent from parents for both years of the study and assent from the students prior to participation.

JUMP Math resources were distributed to teachers in the JUMP group at no cost. To help equate the distribution of new math resources across groups, all of the teachers in the SB2 group received the equivalent of CDN$250 in gift cards to purchase math-related classroom materials of their choosing. Funds were offered after teachers had already consented to participate in the study.

### Materials and methods

#### Participants

Elementary students attending the participating school board were predominantly white; 3% percent were born outside Canada, 5% had a non-English first language and 2% were English language learners (corresponding regional values 11%, 5%, 11%). Twenty-four percent were identified as requiring special education (regional value 19%, see note [50], [51]).

Members of our research team visited the 55 schools that were eligible to participate in the study (they had classes in the study grades and were not listed for potential closure or merger), presented an overview of the study to the school principals and addressed any concerns. Forty-one principals consented to participate. Too many prior commitments were the main reason for declining. Schools with consenting principals were assigned a numerical code and we used a random number table to randomly assign schools to curricula. One school dropped out after random assignment to the SB2 group, hence 40 schools participated (21 JUMP, 19 SB2). This, together with variation in the size of the participating schools, resulted in an imbalanced number of participating teachers and students in the two groups. The percentage of students in the participating schools who reached expectations on the regional math assessment in the year preceding the study was 52% JUMP, 60% SB2 for grade 3 (regional value 68%), and 39% JUMP, 56% SB2 for grade 6 (regional value 58%). [50] Thus, there was room for improvement in students’ math achievement. The average median household income was $68, 556 (range $46, 272 to $86, 181) for the JUMP schools and $68, 503 (range $42, 861 to $86, 791) for the SB2 schools (national median $69, 860). [52]

Teacher eligibility criteria were the same as for the pilot study; accredited to teach in Ontario, in good standing, and not planning to take a leave of absence during the study period. Members of our research team contacted eligible teachers either by email or in person and provided an overview of the study along with a detailed teacher consent form. Questions were encouraged and addressed immediately. In year 1, 115 teachers agreed to take part but 9 (2 JUMP, 7 SB2) declined following random assignment citing overwhelming schedules. Nine teachers who participated (5 JUMP, 4 SB2) left the study in year 1 due either to the end of a contract or for maternity leave and were replaced by new teachers. Thus, there were 106 teachers in year 1; 58 JUMP (31 grade 2, 27 grade 5) and 48 SB2 (26 grade 2, 22 grade 5). In year 2, 92 new teachers agreed to take part but 5 later declined (3 JUMP, 2 SB2) citing overwhelming schedules. However, 20 teachers who participated in year 1 also agreed to participate in year 2 (they taught grade 2 or 5 in year 1 and then inherited study students in grade 3 or 6 in year 2, in the same school). Ten teachers who participated (8 JUMP, 2 SB2) left the study in year 2 for the same reasons cited by teachers who left in year 1, and were replaced by new teachers. Thus, there were 107 teachers in year 2; 59 JUMP (34 grade 3, 25 grade 6) and 48 SB2 (27 grade 3, 21 grade 6). Replacement teachers were recruited to the study immediately and received a combination of the relevant PD and support implementing their assigned curriculum. As teacher changes tended to occur either relatively early or relatively late in the school year, student data were yoked to the predominant teacher and only the data from the predominant teacher were included in our analyses.

Table 4 shows a summary of demographic information for the final sample of teachers. Teachers in the primary division (grades 2 and 3) were mostly female, while in the junior division (grades 5 and 6) the percentage of females ranged from 52 to 65%. Teachers in the JUMP group had somewhat more teaching experience and most teachers in both groups had last studied math in high school (the rest had some university level math, see note [41], [42–44])

**Table 4:** Demographic information for final sample of teachers in Scale-Up RCT.

	Year 1				Year 2			
	Grade 2		Grade 5		Grade 3		Grade 6	
	JUMP	SB2	JUMP	SB2	JUMP	SB2	JUMP	SB2
Number of Teachers	31	26	27	22	34	27	25	21
% Female	94	100	63	59	94	93	60	52
% at least 5 years teaching experience	90	69	81	71	79	70	93	64
% last studied math in high school^a^	87	97	96	95	93	87	88	93

Year 2 includes the 20 teachers who participated in both years of the study.

^a^The remaining teachers had some university level math.

**Fig 2:**
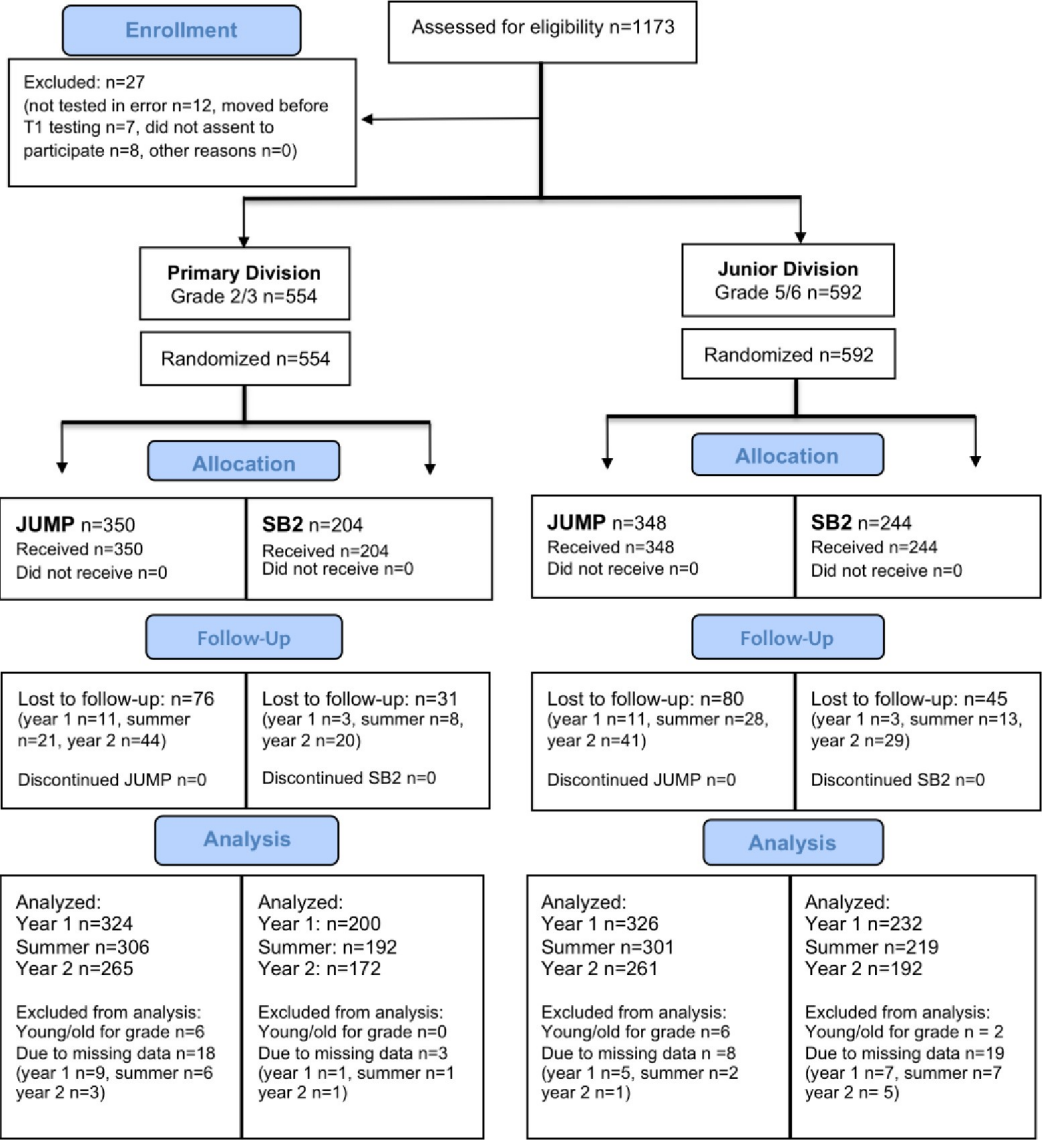
CONSORT flow diagram of student participation in the Scale-Up RCT. Primary students are shown on the left and junior students on the right side of the Fig. See main text for reasons for being lost to follow-up. Young/old for grade denotes students whose date of birth indicated they had started school either a year earlier or a year later than usual. Students were excluded from the analysis for a given time period if they did not have data for either the beginning or the end of that time period, which was determined separately for each outcome measure. The number of students excluded due to missing data shown in the Fig is based on the broad math outcome measure (see measures) but this number varied slightly across the different outcome measures.

Student eligibility criteria was the same as in the pilot study (sufficient grasp of English and no developmental challenges that would preclude full participation, as judged by their teacher) with the exception that both grade 2 and grade 5 students were eligible for the scale-up RCT. Teachers distributed detailed parental consent forms to eligible students in their classes. Fig 2 shows the CONSORT flow diagram of the student participants. Eleven hundred and seventy-three students who met the criteria for eligibility returned signed parental consent forms. Twenty-seven students were excluded from the study; 12 were not tested in error, 8 moved before T1 testing and 7 did not assent to participate. Thus at T1, there were 554 primary students (n = 350 JUMP, 204 SB2) and 592 junior students (n = 348 JUMP, 244 SB2) in the study (see “allocation” in Fig 2). Boys and girls were equally represented. We ran 1000 bootstrap samples of the math fluency data from the pilot study to estimate the power to replicate our findings with the sample size obtained for the scale up study, which was 78.3% and 77.9% for the primary and junior groups, respectively.

Students were considered lost to follow-up after T1 testing if they moved during the study period (either to a school in the other group e.g. a JUMP student who moved to an SB2 school, or to a non-study school) or if the teacher who inherited them in year 2 did not consent to participate in the study (n = 11 teachers, roughly equally distributed across grade and curriculum). The latter group of students (n = 111, primary n = 40 JUMP, n = 15 SB2; junior n = 36 JUMP, n = 20 SB2) were assessed at T3 but not at T4, as parent consent was given for both years. Hence, the greater attrition in year 2 compared to year 1 or the summertime (see “follow-up” in the Fig).

Students whose date of birth indicated they had started school either a year earlier or a year later than usual (which is the year in which children turn 4 years of age, under current policy students cannot be held back or skip a grade once they start school), were considered “young/old for grade” and excluded from the analyses for all time periods (n = 14 students altogether, see Fig 2). Students were also excluded from the analysis for a given time period if they were missing data for either the beginning or the end of that period e.g. students who had data for T1 but not T2 were excluded from the analysis for year 1, of the outcome variable on which they were missing data. The number of students excluded for missing data shown in the Fig is based on the broad math outcome measure, but this number varied slightly across the different outcome measures (see measures and also “analysis” in Fig 2).

The right side of Table 2 shows a summary of the scale-up RCT student characteristics at baseline, which were generally comparable in the two groups (see note [41]), [42–44]. Test scores were in the average to low average range. The primary cohort performed somewhat better than the junior cohort on all of the measures except for working memory, which was more developed in the junior cohort (as expected). The relatively low PSP and CBC scores at baseline (the start of the school year) are to be expected for grade appropriate material.

#### Teacher professional development

Teacher PD in the scale up was the same as in the pilot (1 day in the fall, 1 day mid-year), with some modifications to the delivery of the SB1-PD. Following the fall PD, teachers in the SB2 group expressed concern at the possible bias of having JUMP PD delivered by a well-regarded, external expert in math pedagogy while the SB2-PD was delivered by the board support team. To address their concerns and to help maintain teacher motivation, SB2 teachers in year 1 received an additional ½ day PD in early December, delivered by a well-regarded, external expert in problem-based math instruction, who was nominated by the participating teachers. Accordingly, a different, well-regarded, external expert, also nominated by the teachers, delivered both PD days in year 2. Members of our research team attended all of the SB2-PD sessions, which were in line with problem-based math instruction, and included a review of regional guidelines, implementation guidance, pedagogical demonstrations and small group problem solving.

Note that in addition to the detailed teacher’s guides and student practice and assessment books, the JUMP group teachers also received grade specific JUMP Math smart board materials that were not available at the time of the pilot study.

#### Implementation fidelity

We measured fidelity of implementation of the two curricula in both years of the study by videotaping teachers as they taught their participating class for 1 full math period and then coding the videotapes according to the type of instruction used. Video recording was done around the middle of the school year (after the mid-year PD) in years 1 and 2, by members of the data collection team who were blind to the study hypotheses and to the teacher’s assignment to curricula.

The videotapes were coded by three accredited, occasional (substitute) teachers familiar with the problem-based approach to math instruction and who, apart from coding, were not otherwise involved with the study. They were blind to the study hypotheses and to teacher assignment to curricula. They watched videotapes of the JUMP PD, reviewed the materials provided at the SB2 PD sessions (SB2 trainers declined to be videotaped), received training on how to use the coding scheme and a detailed coding manual for reference. Videotapes were randomly assigned such that each coder coded 1/3 of the videotapes.

Coders watched the entire math class and captured their observations on 3 parallel timelines; teacher activity (what was the teacher doing?), student configuration (how were the students arranged?) and lesson content (what were the students engaged in?). For teacher activity and student configuration, they selected from a set of defined options and indicated start and stop times. The options for teacher activity were: review (of previously taught material), facilitation (asking questions, providing prompts to help students think through the problem), direct instruction (explicit teaching of concepts and procedures, including worked examples), organization (e.g. helping to form groups, find space to work in), circulation (to check on progress), available (attending to the class and addressing queries initiated by students), and distracted (occupied with something not related to the lesson). For student configuration the options were: whole class (students seated at their desks or on the carpet attending to the teacher), small group (working in pairs or small groups) and independent (working individually, usually at their desks). We tallied the total amount of time they observed the teacher in the various activities and the time the students spent in the different configurations, separately, and converted the results to a percentage of the class time, for each teacher. For lesson content, the coder noted the specific activity the students were engaged in (e.g. doing a fact fluency exercise, working on a problem), indicating start and stop times.

Coders then reviewed their observations and made 3 judgments: 1. They indicated the curriculum with which the lesson was most aligned selecting from: problem-based learning (SB2), guided discovery and scaffolding (JUMP), or neither, 2. They indicated the degree to which the observed lesson was aligned with their selected curriculum on a scale from 1-not at all to 7-very well aligned, and 3. They rated their confidence in their choice of curriculum from 1-not very confident to 5-very confident.

Twenty percent of each coder’s assignment was randomly selected and then randomly distributed to the 2 remaining coders for second coding. Cohen’s Kappa for overall inter-rater agreement was κ = .72, *p* < .0001. Kappa values of .61 -.80 indicate substantial agreement while values greater than .81 indicate almost perfect agreement [53, 54]. When they agreed about curriculum, their alignment ratings were identical or within 1-point 87% of the time and within 2 points 13% of the time. Disagreements regarding type of instruction were discussed as soon as possible after second coding but only the data from the original coder were used in our analyses. [see also S1 Text].

Table 5 summarizes the rate of participation in the observations/videotaping. One hundred and sixty four teachers consented to the videotaping, 16 of who participated and were videotaped in both years of the study (180 videotapes altogether, 85% of the participating classes). As the table shows, the rate of participation, though generally very good, was higher in year 1 than in year 2, especially for the junior teachers (see note [41]), [42–44].

**Table 5:** Summary of teacher participation in observations/videotaping.

	Year 1				Year 2				
	Grade 2		Grade 5		Grade 3		Grade 6		
Assigned Curriculum	JUMP	SB2	JUMP	SB2	JUMP	SB2	JUMP	SB2	Total
Number of participating teachers ^a^	31	26	27	22	34	27	25	21	213
**%** (number) of teachers observed	**94** (29)	**88** (23)	**96** (26)	**100**(22)	**82** (28)	**78** (21)	**68** (17)	**67** (14)	**85** (180)
**%** (number) of observed teachers with assigned-observed curriculum congruence ^b^	**90** (26)	**87** (20)	**92** (24)	**86** (19)	**93** (26)	**81** (17)	**88** (15)	**64** (9)	**87** (156)
**%** (number) of congruent teachers with degree of alignment with curriculum ≥ 5 on 7-point scale, higher = more aligned	**88** (23)	**85** (17)	**71** (17)	**95** (18)	**77** (20)	**82** (14)	**67** (10)	**56** (5)	**79** (124)
**%** (number) of congruent teachers with coder confidence in type of instruction ≥ 4 on 5-point scale, higher = more confident	**88** (23)	**90** (18)	**85** (22)	**100**(19)	**96** (25)	**88** (15)	**93** (14)	**100** (9)	**93** (145)
**%** (number) of observed teachers with high fidelity (curriculum congruence, high alignment and high confidence)	**76** (22)	**74** (17)	**65** (17)	**82** (18)	**68** (19)	**67** (14)	**59** (10)	**36** (5)	**68** (122)

^a^ Year 2 values include teachers who participated in both years of the study and agreed to be observed (n = 16).

^b^Only 1 teacher (grade 3, SB2) was coded as using neither the JUMP nor the SB2 curriculum. Coder confidence rating for this observation was 4 on a 5-point scale (higher scores indicate greater confidence).

The large majority of observed teachers were coded as using their assigned curriculum, but a few teachers were coded as using the other curriculum (i.e. JUMP teachers using SB2 methods, or vice versa). These incongruent cases were roughly equally distributed across grade and condition (2–3 cases each or 7–14% of the observed classes) with the exception that for the SB2 group in grade 6, 5 of the observed classes (36%) were coded as JUMP lessons (by contrast only 12% of grade 6 JUMP classes were coded as SB2 lessons). When the assigned and observed curriculum were congruent, coder’s judgments of degree of alignment with type of instruction and their confidence in their ratings was generally good; for the majority of cases alignment was at least 5 out of 7 and confidence at least 4 out of 5. Teachers who met these three criteria—assigned/observed curriculum congruence, high alignment and high coder confidence—were considered high implementation fidelity teachers.

Of note, in the cases of curriculum congruence, degree of alignment and coder confidence did not differ significantly from year to year for either group of primary teachers or for the junior JUMP teachers (primary mean alignment JUMP 5.3 year 1 and 4.9 year 2, SB2 5.4 both years; primary mean confidence JUMP 4.2 both years, SB2 4.3 year 1, 4.1 year 2; junior mean alignment JUMP 5.1 year 1 and 4.8 year 2, mean confidence 4.3 year 1, 4.1 year 2). However, for the junior SB2 teachers, alignment was significantly higher in year 1 compared to year 2, but coder confidence did not differ from year to year (mean alignment 5.5 year 1 and 4.7 year 2, *p* = .04, mean confidence 4.1 in both years; all comparisons based on Wilcoxon rank sum tests).

**Fig 3:**
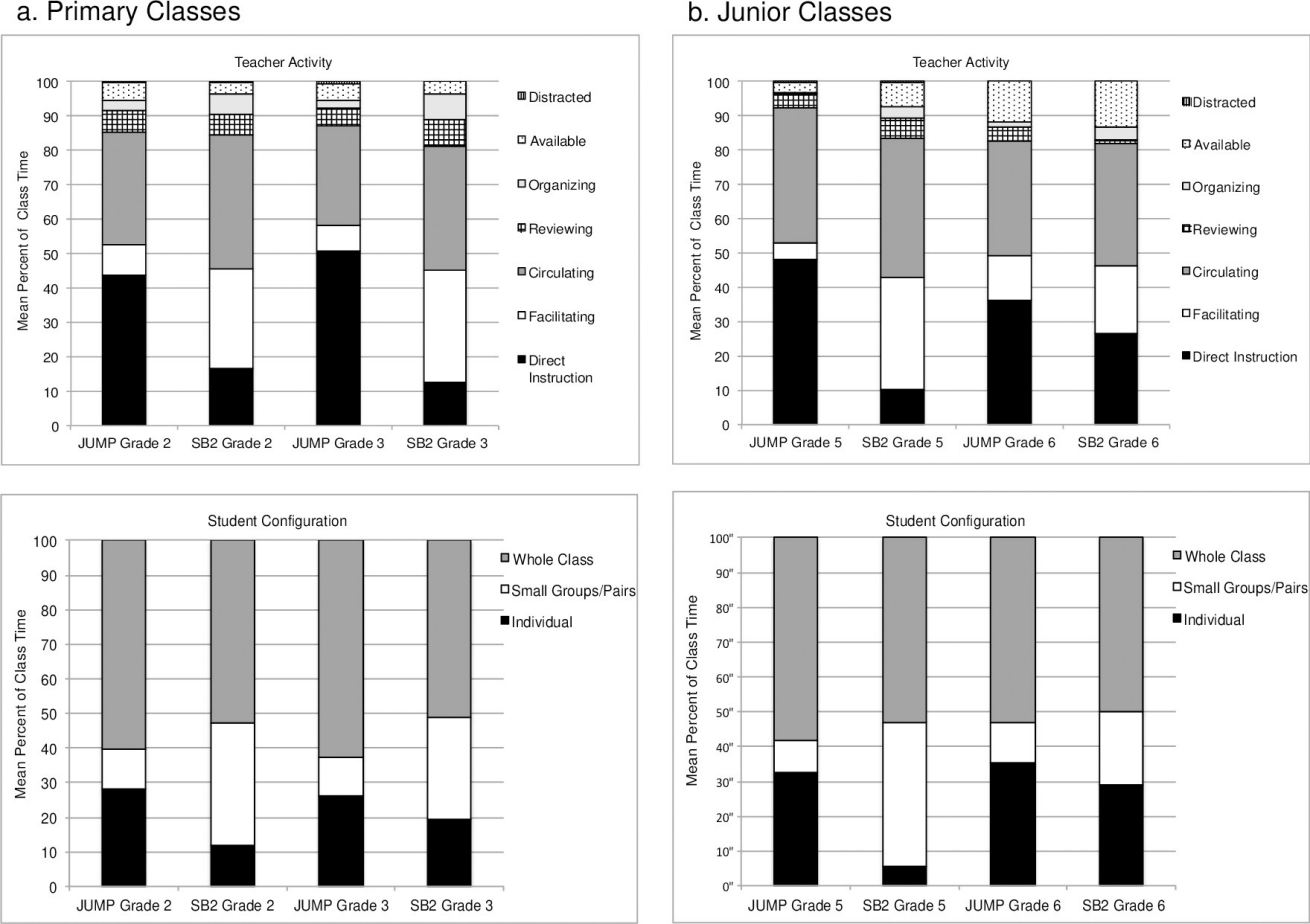
Distribution of time spent on teacher activities and in different student configurations in observed classes.

Fig 3 shows the distribution of mean time spent in the different teacher roles and student configurations, for all of the observed classes (see also Table E in S1 Text). The results were generally consistent with what we would expect for the different curricula. For the primary classes (left side of Fig 3), the JUMP teachers spent substantially more time on direct instruction and relatively little time facilitating, while the pattern for the SB2 teachers was reversed, both groups spent a substantial amount of time circulating and some time on review. The JUMP students spent relatively more time working independently and less time working in small groups, while for the SB2 students this pattern was reversed. In both groups, the whole class was taught together at least half of the time, which reflects the time spent on direct instruction and discussion distributed throughout the JUMP lesson and the time spent on the problem-presentation at the outset and then reconvening at the end of the SB2 lesson.

The pattern we observed for the primary classes also held for the junior classes (right side of Fig 3), for both groups in grade 5 and for the JUMP group in grade 6. However, the SB2 lessons in grade 6 appeared more similar to, than distinct from, the JUMP lessons in that teachers spent relatively more time on direct instruction and less time facilitating and their students spent somewhat more time working individually and less time working in small groups. These findings, together with the relatively small number of SB2 teachers who participated in the observations in grade 6 (14) and the even smaller number with assigned/observed curriculum congruence (9 out of 14) raise concerns regarding fidelity of program delivery for the SB2 group in grade 6, and therefore suggest caution in interpreting the results for the junior student outcomes in year 2.

#### Measures and procedures

**Math achievement**

Our primary math achievement measures included the math fluency and calculation scales (two computational measures also used in the pilot study), as well as the applied problems scale from the WJ-III, which together yield a broad math cluster score. (see S1 Text). We supplemented these standardized tests with another problem solving and another computation measure. The supplementary measures comprise grade appropriate material only and give credit for appropriate steps taken toward the correct solution as well as the correct solution, providing additional opportunities for children to demonstrate the extent of their mathematical knowledge.

The problem-solving process measure (PSP), is a paper and pencil test based on items from a bank of word problems that have been used on the regional math assessment written annually at the end of grades 3 and 6. We developed different 5-problem tests for each of the study grades (2, 3, 5 and 6) with equivalent fall and spring forms of each test. Children were required to show their work as well as their final answer. PSP and Applied Problems from the WJ-III were significantly correlated at T1 (baseline), r = .61 and .52 for grade 2 and 5, respectively, and also at T4 (end of year 2), r = .59 and .64 for grade 3 and grade 6, respectively, all *p*’s < .0001. We also developed detailed scoring manuals for each grade based on publicly available guidelines. Children received a score between 0 and 40 for each of the 5 questions. Test scores represent the mean of the 5 question scores. (see S1 Text for further details regarding test development and scoring).

Curriculum-based computation (CBC) is a timed, paper and pencil test from the Monitoring Basic Skills Progress math kit [55] comprising 25 grade-specific computation questions. Children received a different but equivalent form of the test at the fall and spring testing. Scoring followed the developer’s instructions; 1 point was awarded for every digit in the correct position in the final answer. E.g. if the answer was 18, children received 2 points for 18, 1 point for 17 and 0 points for 81. Total scores for each test were converted to a percentage of the possible points on that test.

**Additional measures**

We measured reading achievement with the broad reading cluster from the WJ-III, which is based on performance on letter-word identification, reading fluency and passage comprehension.

We assessed IQ and working memory using the same measures used in the pilot study. Finally we assessed processing speed with the digit and letter versions of the Rapid Automatized Naming Test. [56] We assessed math and reading achievement at all four data collection time points but IQ, working memory and processing speed at baseline only. (see S1 Text)

Teachers completed a questionnaire probing their teaching experience, highest level of math education and the amount of math instruction time per week provided to their participating class. They also rated their math teaching experience during the study period on a 10-point scale from “very poor” (0) to “excellent” (10).

#### Procedures

Children were tested either individually or in small groups in a quiet area in their school. Testing was done by a local team of accredited, occasional (substitute) teachers who were familiar with the school board, but blind to the study hypotheses and to random assignment to curricula. The testers did not participate in the study and did not teach in the participating schools during the study period. The study measures were administered in 3 sessions that took place on separate days. Breaks were given as needed.

### Analytic plan

As we were interested in comparing progress in math achievement between the two groups, within each of the three time periods: year 1 (T1 to T2); summer (T2 to T3); and year 2 (T3 to T4), growth in achievement was modeled using repeated measures hierarchical regression analysis, with repeated measures on students nested within classrooms, using PROC MIXED in SAS 9.4 (copyright 2002–2012 SAS Institute Inc., Cary, NC, USA). Classrooms nested in schools was not included in the models as the number of participating teachers was imbalanced across schools and there was often only one participating teacher in the study grades in a school. For PSP and CBC, we omitted the summer period as the test content was grade specific. We entered baseline performance on the outcome variable, broad reading (at baseline), sex, age on the first day of school, inter-test interval (days), curriculum (JUMP, SB2) and time period (year 1, summer, year 2), as well as 2-way interactions of inter-test interval by time period, baseline by time period, and curriculum by time period, to yield the following model:

Y_ijkt_ = μ + *b*_i_ + α_j_ + δ_t_ + (*a*δ)_it_ + β_1t_*X*_1tk_ + β_2t_*X*_2k_ + β_3_*X*_3k_ + β_4_*X*_4k_ + β_5_*X*_5k_ + *e*_ijkt_

μ Denotes the intercept

*b*_*i*_ Random deviation of the *i*^th^ (classroom) teacher’s intercept from μ, *b*_i_ ~ *N*(0,σ_b_^2^)

*α*_j_ Effect for condition *j*

δ_t_ Effect for time period *t*

(*a*δ)_jt_ Condition × time period interaction

β_1t_ Denotes the effect of the duration (days) of time period *t*, at time period *t* (all duration

× time period effects)

β_2t_ Denotes the effect of baseline math score on the effect of time period *t* (all baseline

math score × time period effects)

β_3_ Denotes the effect of baseline reading score

β_4_ Denotes the effect of age on September 3 of year 1

β_5_ Denotes the effect of sex

*X*_1tk_ Duration in days of time period *t*, child *k*

*X*_2k_ Baseline math score for child *k*

*X*_3k_ Baseline reading score for child *k*

*X*_4k_ Age on September 3 of year 1, child *k*

*X*_5k_ Indicator variable for sex, child *k*

*e*_*ij*kt_ Random error associated with child *k* at time *t*, in classroom *i* assigned to condition *j*

Var(*e*_*ij*kt_) = σ_t_^2^ and Cov[*e*_*ij*kt,_ *e*_*ij*kt’_] = σ_kk’_

To address our main research question regarding the impact of curriculum on math progress, we focus on the effect of curricula in each of the three time periods (see note [57]). Following similar previous research (see e.g. [58]), and to simplify the models, we analyzed the data for the two age groups (primary, grades 2/3; junior, grades 5/6) separately. Effect sizes were derived from estimates from the hierarchical repeated measures models, as in the pilot study.

We first report the results for the “per protocol” analysis (PP) i.e. based on all participating students, according to their randomly assigned curriculum. Note that we were unable to carry out intent-to-treat analysis i.e. including all participants randomly assigned to the two groups, regardless of whether they received the treatment or not—which is normally required for a clinical trial—as we did not recruit (or assess) any students at the 1 SB2 school that dropped out of the study after random assignment. As an important objective of the scale up RCT was to replicate and extend the findings from the pilot study, we report the results for the junior students first followed by the results for the primary students. Furthermore, for each age group, we report the results for the main outcomes–the WJ-III math achievement scales–followed by the results for the supplementary math outcomes. (see note [59]), [60]

Finally, based on our findings for implementation fidelity, including the finding that assigned and observed curriculum were sometimes incongruent, we also analyzed the data from only those students whose teachers were observed and met the criteria for high fidelity instruction (HiFi; see the results for implementation fidelity). For these analyses, year 1 and summer results are based on the data from students who had HiFi teachers in year 1, and year 2 results are based on data from students who had HiFi teachers in year 1 and year 2. The number of primary students who met these criteria was 256 JUMP and 117 SB2 for the analyses for year 1 and the summer, and 124 JUMP and 70 SB2 for the analyses for year 2. Note that these HiFi analyses were only carried out for the primary students. The more limited number of junior teachers who participated in the observations and even smaller number who met the criteria for high fidelity, especially in the SB2 group in year 2, precluded any meaningful analysis for the junior HiFi students.

### Results

#### Per protocol (PP) analysis

**Junior students: main outcomes**

**Fig 4:**
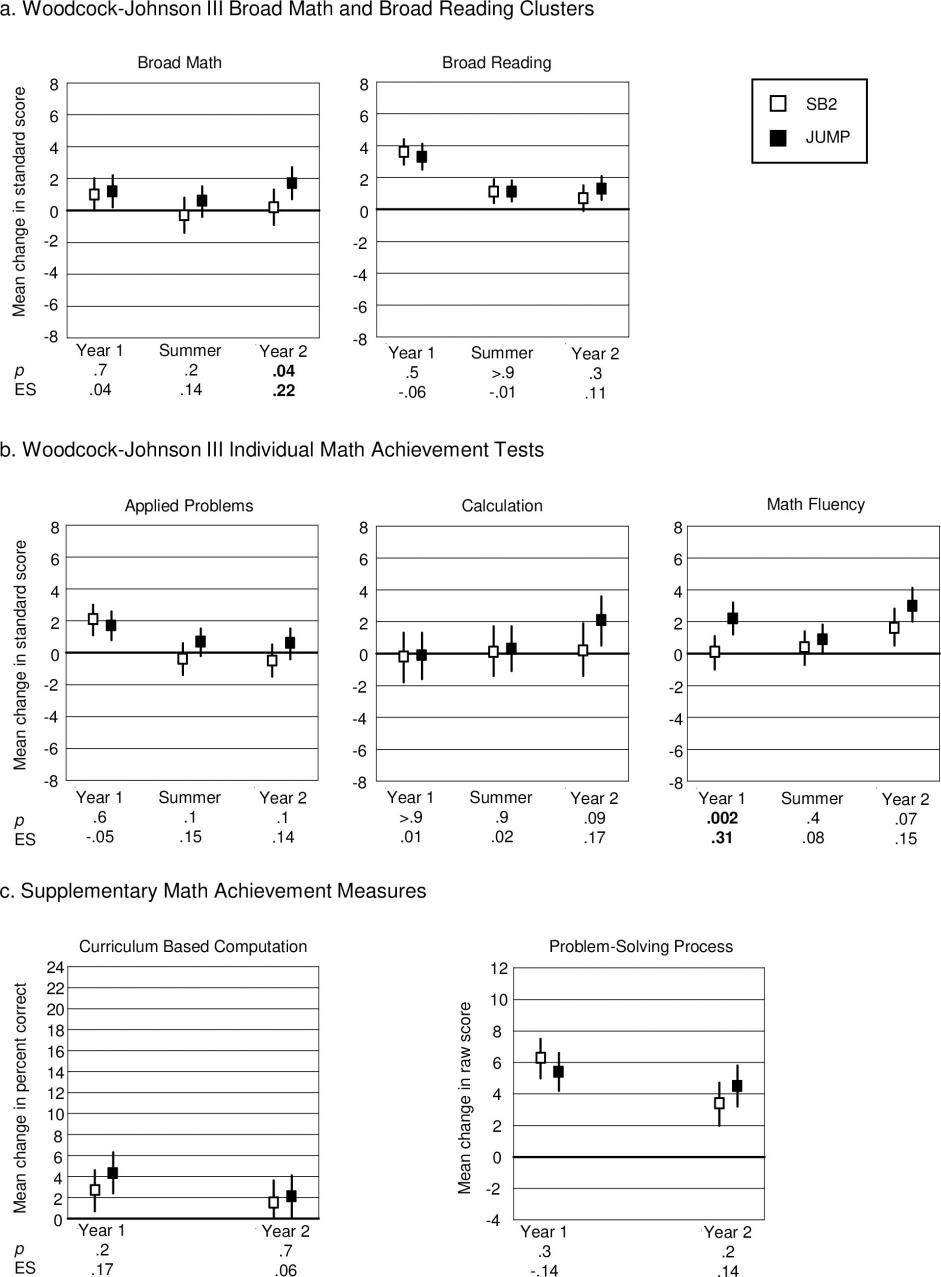
Results for the Junior Students. Broad Math (left side of panel a) is based on performance on applied problems, calculation and math fluency (shown separately in panel b). Vertical lines indicate 95% confidence limits around mean change scores. *P*-values and effect sizes (ES) in the Fig are for the difference between the group means. Results for the Woodcock-Johnson III measures are based on standard scores and therefore indicate progress relative to same aged peers, which is represented by the 0 line. Vertical lines that *do not* intersect the 0 line indicate mean change that is significantly different from expected change, based on test norms for the standardized measures (panels a and b) and 0 change for the supplementary measures (panel c).

Fig 4 shows the results for the PP analysis for the junior students. In general, progress in math achievement for the junior students was fairly stable across the three time periods. Comparing progress between the groups we found that, for broad math, the two groups did not differ significantly in year 1 or the summer. However, the JUMP group made significantly greater gains than the SB2 group in year 2 (1.0 SB2, 1.2 JUMP, *p* = .7, ES = .04, year 1; -0.3 SB2, 0.6 JUMP, *p* = .2, ES = .14, summer; 0.2 SB2, 1.7 JUMP, *p* = .04, ES = .22, year 2). In contrast, for broad reading there were no significant differences between the two groups in any time period (3.6 SB2, 3.3 JUMP, *p* = .5, ES = -.06, year 1; 1.1 both groups, *p* > .9, ES = -.01, summer; 0.7 SB2, 1.3 JUMP, *p* = .3, ES = 0.11, year 2; see Fig 4, panel a).

These findings were generally corroborated when we compared observed gains to expected gains based on test norms. Although only the JUMP group made significantly greater than expected progress on broad math in year 1 (*p* = .06 SB2, *p* = .01 JUMP), progress in both groups was not significantly different from expected in the summertime (*p* = .6 SB2, *p* = .2 JUMP). Furthermore, only the JUMP group continued to make significantly greater than expected progress in year 2 (*p* = .7 SB2, *p* = .001 JUMP). For broad reading, both groups made greater than expected progress in all time periods, except for the SB2 group in year 2 (*p* < .0001, *p* = .004 and *p* = .07 SB2; *p* < .0001, *p* = .001, and p < .0005 JUMP; for year 1, the summer, and year 2, respectively).

Analyses of the individual standardized tests (Fig 4 panel b) showed that the JUMP curriculum had a positive impact on math fluency, where significantly greater gains were already apparent in year 1 (0.1 SB2, 2.2 JUMP, *p* = .002, ES = .31). The group difference was not significant in year 2, (1.6 SB2, 3.0 JUMP, *p* = .07, ES = .15). The difference between groups was not significant in either year, for calculation (-0.2 SB2, -0.1 JUMP, *p* > 0.9, ES = .01 year 1; 0.2 SB2, 2.1 JUMP, *p* = .09, ES = .17 year 2) or for applied problems (2.1 SB2, 1.7 JUMP, *p* = .6, ES = -.05 year 1; -0.5 SB2, 0.6 JUMP, *p* = .1, ES = .14 year 2), and there were no significant differences between the two groups in gains on any of these measures in the summertime (0.4 SB2, 0.9 JUMP, *p* = .4, ES = .08 math fluency; 0.1 SB2, 0.3 JUMP, *p* = .9, ES = .02 calculation; -0.4 SB2, 0.7 JUMP, *p* = .1, ES = .15 applied problems).

Comparing observed to expected gains we found that the JUMP group made significantly greater than expected gains on math fluency in all time periods (*p*’s < .0001 years 1 and 2, *p* = .05 in the summer), while the SB2 group’s gains were only significantly greater than expected in year 2 (*p* = .9 year 1, *p* = .5 summer, *p* = .006 year 2). For calculation, gains in both groups were not significantly different from expected gains in year 1 or the summer (*p* = .8 SB2, *p* = .9 JUMP year 1; *p* = .9 SB2, *p* = .7 JUMP summer) and only the JUMP group made significantly greater than expected gains in year 2 (*p* = .8 SB2, *p* = .008 JUMP). For applied problems, both groups made significantly greater than expected gains in year 1 (*p* < .0001 SB2, *p* = .0002 JUMP) but their gains were not significantly different from expected gains in the summer or in year 2 (*p* = .5 SB2, *p* = .1 JUMP summer; *p* = .3 SB2, *p* = .2 JUMP year 2).

**Junior students: supplementary outcomes**

For PSP and CBC there were no significant group differences in either year (PSP 6.3 SB2, 5.4 JUMP, *p* = .3, ES = -.14 year 1; 3.4 SB2, 4.5 JUMP, *p* = .2, ES = .14 year 2; CBC 2.7 SB2, 4.3 JUMP, *p* = .2, ES = .17 year 1; 1.5 SB2, 2.1 JUMP, *p* = .7, ES = .06 year 2, see Fig 4 panel c). For PSP, both groups made significant gains (greater than 0) in both years (all *p*’s < .0001). However, for CBC, whereas both groups made significant gains (greater than 0) in year 1 (*p* = .009 SB2, *p* < .0001 JUMP), only the JUMP group made significant gains in year 2 (*p* = .2 SB2, *p* = .04 JUMP).

**Primary students: main outcomes**

**Fig 5:**
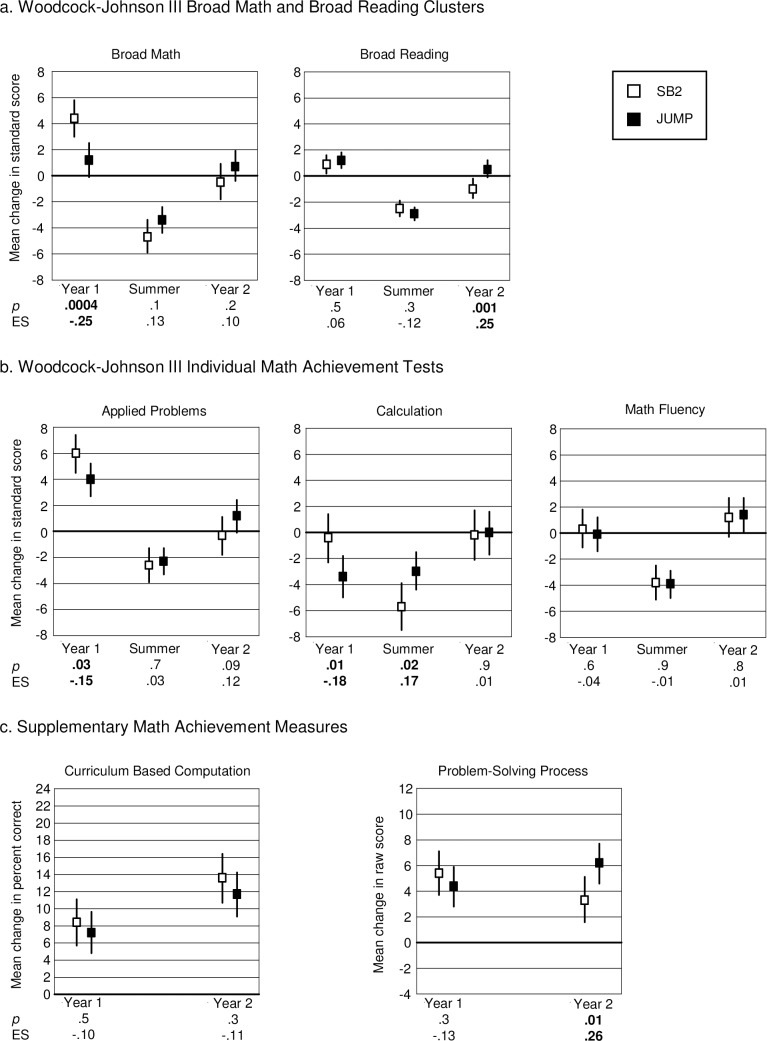
Results for the primary students. Broad Math (left side of panel a) is based on performance on applied problems, calculation and math fluency (shown separately in panel b). Vertical lines indicate 95% confidence limits around mean change scores. *P*-values and effect sizes (ES) in the Fig are for the difference between the group means. Results for the Woodcock-Johnson III measures are based on standard scores and therefore indicate progress relative to same aged peers, which is represented by the 0 line. Vertical lines that *do not* intersect the 0 line indicate mean change that is significantly different from expect change, based on test norms for the standardized measures (panels a and b) and 0 change for the supplementary measures (panel c).

Fig 5 shows the results for the PP analysis for the primary students. Progress in math achievement was generally less stable over the three time periods for the primary students compared to the junior students. As the Fig for broad math shows, the SB2 group made significantly greater progress in year 1 (4.4 SB2, 1.2 JUMP, *p* = .0004, ES = -.25) but there were no significant differences in progress in the two groups either in the summer or in year 2 (-4.7 SB2, -3.4 JUMP, *p* = .1, ES = .13 summer; -0.5 SB2, 0.7 JUMP, *p* = .2, ES = .10 year 2). For broad reading, there were no significant differences in progress between the groups either in year 1 or the summer (0.9 SB2, 1.2 JUMP, *p* = .5, ES = .06 year 1; -2.5 SB2, -2.9 JUMP, *p* = .3, ES = -.12 summer), but the JUMP group experienced significantly greater progress in year 2 (-1.0 SB2, 0.5 JUMP, *p* = .001, ES = .25; see Fig 5 panel a).

These results were corroborated when we compared progress within each group to expected progress based on test norms. For broad math, both groups made significantly greater than expected gains in year 1 (*p* < .0001 SB2, *p* = .05 JUMP), lost significant ground in the summer (*p*’s < .0001), and their gains were not significantly different from expected gains in year 2 (*p* = .5 SB2, *p* = .2 JUMP). For broad reading, both groups also made significantly greater than expected gains in year 1 (*p* = .02 SB2, *p* = .0003 JUMP), and lost significant ground in the summer (*p*’s < .0001), but whereas the SB2 group also made significantly less than expected gains (*p* = .01), the JUMP group gains were not significantly different from expected gains, in year 2 (*p* = .1).

Analyses of the individual WJ-III math achievement tests (Fig 5 panel b) indicated that the pattern of results for broad math was largely due to performance on applied problems and calculation. For applied problems, the SB2 group gained significantly more than the JUMP group in year 1 (6.0 SB2, 4.0 JUMP, *p* = .03, ES = -.15) but progress in the two groups was not significantly different either in the summer or in year 2 (-2.6 SB2, -2.3 JUMP, *p* = .7, ES = .03 summer; -0.3 SB2, 1.2 JUMP, *p* = .09, ES = .12 year 2). In both groups, growth on calculation was generally more modest compared to applied problems, especially in year 1. The SB2 group made significantly greater progress in year 1 (-.4 SB2, -3.4 JUMP *p* = .01, ES = -.18), but lost significantly more ground in the summertime (-5.7 SB2, -3.0 JUMP, *p* = .02, ES = .17), compared to their JUMP peers. Progress in the two groups was not significantly different in year 2 (-.2 SB2, 0.0 JUMP, *p* = .9, ES = .01). For Math Fluency, there were no significant differences between the two groups in any time period (0.3 SB2, -0.1 JUMP, *p* = .6, ES = -.04 year 1; -3.8 SB2, -3.9 JUMP, *p* = .9, ES = -.01 summer; 1.2 SB2, 1.4 JUMP, *p* = .8, ES = .01 year 2).

Comparing performance within groups to expected growth based on test norms corroborated the between group findings. For applied problems, both groups gained significantly more than expected in year 1 (*p*’s < .0001) and significantly less than expected in the summertime (*p*’s < .0001). Neither group’s progress was significantly different from expected progress in year 2 (*p* = .6 SB2, *p* = .07 JUMP). For calculation, the SB2 group gains were not significantly different from expected, while the JUMP group gains were significantly less than expected in year 1 (*p* = .6 SB2, *p* = .0001 JUMP) and both groups gains were significantly less than expected in the summer (*p* < .0001 SB2; *p* = .0001 JUMP). Gains in both groups were not significantly different from expected gains in year 2 (*p* = .8 SB2, *p* > .9 JUMP). For math fluency, both groups progressed as much as expected in year 1 (*p* = .6 SB2, *p* = .8 JUMP) and significantly less than expected in the summer (*p*’s < .0001). Only the JUMP group progressed significantly more than expected in year 2 (*p* = .1 SB2, *p* = .03 JUMP).

**Primary students: Supplementary outcomes**

For PSP, the difference between groups was not significant in year 1 (5.4 SB2, 4.4 JUMP, *p* = .3, ES = -.13) but the JUMP group gained significantly more than the SB2 group in year 2 (3.3 SB2, 6.2 JUMP, *p* = .01, ES = .26). For CBC, there were no significant differences between groups in either year (8.4 SB2 8.4, 7.2 JUMP, *p* = .5, ES = -.10 year 1; 13.6 SB2, 11.7 JUMP, *p* = .3, ES = -.11 year 2, see Fig 5 panel c). All of the gains for PSP and CBC were significantly greater than zero (PSP both *p* ‘s < .0001 year 1; *p* = .0003 SB2, *p* < .0001 JUMP year 2; CBC all *p*’s < .0001).

#### High fidelity (HiFi) analysis

**Primary students: Main outcomes**

**Fig 6:**
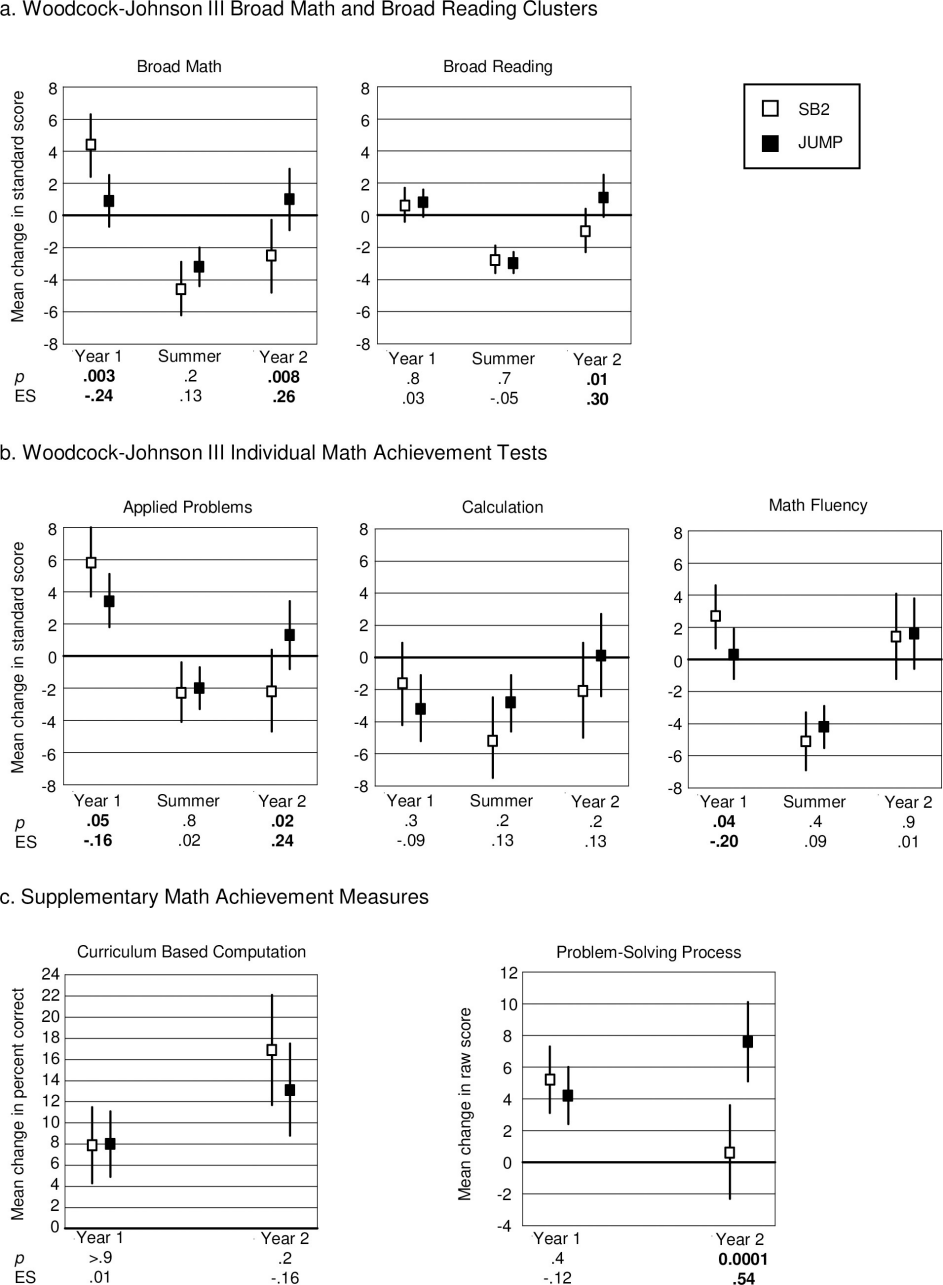
Results for the high fidelity primary students. Results for students who received high fidelity instruction. Broad Math (left side of panel a) is based on performance on applied problems, calculation and math fluency (shown separately in panel b). Vertical lines indicate 95% confidence limits around mean change scores. *P*-values and effect sizes in the Fig are for the difference between the group means. Results for the Woodcock-Johnson III measures are based on standard scores and therefore indicate progress relative to same aged peers, which is represented by the 0 line. Vertical lines that *do not* intersect the 0 line indicate mean change that is significantly different from expected change, based on test norms for the standardized measures (panels a and b) and 0 change for the supplementary measures (panel c).

The results for the primary HiFi students are shown in Fig 6. As the Fig shows, the overall pattern of findings was in line with the results obtained for the PP primary sample, with the differences between groups generally becoming more pronounced. For broad math, there was a significant advantage to the SB2 group in year 1 (4.4 SB2, 0.9 JUMP, *p* = .003, ES = -.24), but the JUMP group progressed significantly more in year 2 (-2.5 SB2, 1.00 JUMP; *p* = .008, ES = .26). There was no significant difference in progress between the groups in the summertime (-4.6 SB2, -3.2 JUMP, *p* = .2, ES = .13). In contrast, for broad reading, there were no significant differences between the groups in year 1 (0.6 SB2, 0.8 JUMP, *p* = .8, ES = .03) or the summer (-2.8 SB2, -3.0 JUMP, *p* = .7, ES = -.05), but the JUMP group made significantly greater progress in year 2 (-1.0 SB2, 1.1 JUMP, *p* = .01, ES = .30; see Fig 6 panel a).

As before, this pattern of findings was corroborated when we compared within group performance to expected progress based on test norms. For broad math, the SB2 group progressed significantly more than expected in year 1 (*p* < .0001) and less than expected in year 2 (*p* < .03), while the JUMP group progress was not significantly different from expected in either year (both *p*’s = .3). Both groups progressed significantly less than expected in the summer (*p*’s < .0001). For broad reading, neither group’s gains were significantly different from expected, in either year (*p* = .2 SB2, *p* = .07 JUMP for both years).

The results for broad math for the HiFi sample reflect the results for all three WJ-III math achievement measures. In year 1, the SB2 group made significantly more progress than the JUMP group on applied problems (5.8 SB2, 3.4 JUMP, *p* = .05, ES = -.16) and math fluency (2.7 SB2, 0.3 JUMP *p* = .04, ES = -.20) but not on calculation (-1.6 SB2, -3.2 JUMP, *p* = .3, ES = -.09). However, in year 2, the JUMP group made significantly greater gains on applied problems (-2.2 SB2, 1.3 JUMP, *p* = .02, ES = 0.24) and there were no significant differences between the groups on either math fluency (1.4 SB2, 1.6 JUMP, *p* = .9, ES = .01) or calculation (-2.1 SB2, 0.1 JUMP, *p* = .2, ES = .13; see Fig 6 panel b).

Comparing within group progress to expected growth we found that for applied problems, both groups improved significantly more than expected in year 1 (*p* < .0001 SB2, *p* = .0001 JUMP), significantly less than expected in the summer (*p* = .02 SB2 and *p* = .003 JUMP) and their progress was not significantly different from expected in year 2 (*p* = .09 SB2, *p* = .2 JUMP). For math fluency, the SB2 group advanced significantly more than expected in year 1 (*p* = .009) and as much as expected in year 2 (*p* = .3), while the JUMP group advanced as much as expected in both years (*p* = .7 year1, *p* = .2 year 2). Both groups advanced significantly less than expected in the summer (*p*’s < .0001). For calculation, progress in the SB2 group was not significantly different from expected in both years and significantly less than expected in the summer (*p*’s = .2 year 1 and year 2, *p* < .0001 summer). The JUMP group progressed significantly less than expected in year 1 and in the summer but grew as much as expected in year 2 (*p* = .003 year 1, *p* = .002 summer, *p* = .9 year 2).

**Primary students: supplementary outcomes**

The results for PSP and CBC for the HiFi primary sample were also in line with the results for the PP primary sample. For PSP, the groups did not differ significantly in year 1 (5.2 SB2, 4.2 JUMP, *p* = .4, ES = -.12) but the JUMP group grew significantly and substantially more than the SB2 group in year 2 (0.6 SB2, 7.6 JUMP, *p* = .0001, ES = .54). For CBC, progress in the two groups did not differ significantly in either year (7.9 SB2, 8.00 JUMP, *p* >.9, ES = .01 year 1; 16.9 SB2, 13.1 JUMP, *p* = .2, ES = -.16 year 2, see Fig 6 panel c). Finally, for PSP, both groups made significant gains in year 1 and only the JUMP group made significant gains in year 2 (significant *p*’s < .0001, *p* = .7 SB2 year 2), and for CBC both groups made significant gains in both years (all *p*’s < .0001).

#### Teachers

Teachers’ reports of their experience teaching math in the study year (s) were generally favourable in both groups and across study grades and none of the differences between groups were significant (based on Wilcoxon rank sum tests; *M* grade 2: 7.8 SB2, 7.1 JUMP; grade 3: 7.4 SB2, 7.5 JUMP; grade 5: 7.7 SB2, 8.0 JUMP; grade 6: 7.3 SB2, 6.6 JUMP).

### Discussion

#### Junior students

Performance by the junior students was fairly stable across the three time periods. Even in the summer break when students usually experience losses [39], both groups’ progress was in keeping with that of same aged peers (based on available norms).

A key finding for the junior students was that we replicated the positive effect of JUMP instruction on math fluency that we found in the pilot study. These effects were already apparent in year 1 when the JUMP group made significantly greater gains than the SB2 group and only the JUMP group made significantly greater than expected gains based on available norms. It is possible that the advantage to the JUMP group on math fluency in year 1 facilitated their greater overall progress, as shown by significantly greater progress on broad math, in year 2.

Indeed, the JUMP group’s greater growth on broad math in year 2 is notable given our findings regarding implementation fidelity suggesting somewhat diluted program delivery in the SB2 group in year 2. Although the participation rate for the observations was comparable in the JUMP (68%) and SB2 groups (67%) in year 2, only 12% of the observed JUMP teachers were coded as using the SB2 group methods whereas 36% of the observed SB2 teachers who were coded as using JUMP instruction. Moreover, amongst those observed SB2 teachers who used their assigned method of instruction, almost half had relatively low alignment scores (less than 5 on a 7-point scale). As a group, the SB2 teachers spent relatively more time on direct instruction and less on facilitation, and their students spent more time working individually than in small groups, which is more similar to what we would expect to see in a JUMP class. These departures from SB2 instruction may have contributed to the finding that the SB2 students also progressed significantly more than expected on math fluency in year 2 (but not in year 1 or the summertime, based on test norms) and hence that the difference between the groups in year 2 was not significant.

We did not find a notable JUMP advantage on CBC, another computation measure, but this could be due to an apparent floor effect. Both groups made significant progress in year 1 and only the JUMP group made significant progress in year 2. But scores in both groups were very low at baseline and overall gains in both groups were limited. We vetted the CBC measure with non-study teachers prior to the scale-up study but it remains possible that the grade 5/6 Canadian curriculum is not optimally aligned with the content of the grade 5/6 CBC, which is an American measure.

A surprising finding was that the SB2 group did not fare significantly better than the JUMP group on either measure of problem solving, in any time period. Indeed, the SB2 group did not show any significant advantages even in year 1 when both teachers and students in JUMP classrooms were new to JUMP (which might have slowed progress temporarily), and in the face of high implementation fidelity. We observed all but 1 of the participating grade 5 teachers in year 1 and the large majority was using their randomly assigned curriculum with high alignment and coder confidence.

Finally, as in the pilot study, the finding that there were no group differences on broad reading in the scale up study (in any time period) suggests that, for the junior students, the significant effects of JUMP instruction were specific to math learning.

#### Primary students

A different pattern of results emerged for the primary students. In general, math progress was less stable for the primary students compared to their junior counterparts, with both groups losing substantial ground in the summertime when the students likely did not receive formal instruction. Furthermore, whereas the SB2 students made greater progress overall in year 1, the JUMP students experienced greater gains, on some measures, in year 2. This pattern of results became more pronounced when we restricted the analyses to only those students whose teachers were observed and who received high fidelity instruction.

The key finding for the primary students was an effect of curriculum on problem solving that varied from year to year. Although both groups made substantial gains, the SB2 group significantly outperformed their JUMP peers on applied problems in year 1 (a finding that obtained in both the PP and HiFi samples). However, the advantage to the SB2 group did not persist. Gains in the two groups did not differ significantly in the summertime for either the PP or the HiFi samples nor for the PP sample in year 2, and it is worth mentioning that we found significantly greater gains in the JUMP for the HiFi sample in year 2. The two groups made similar gains on PSP in year 1 but here as well the JUMP group made significantly greater gains than the SB2 group in year 2 (for both the PP and HiFi samples).

The change in direction of the group means from year to year along with our results for implementation fidelity suggest that, for primary students, the benefits of JUMP for problem solving may take time to manifest. In the first year, both the JUMP group teachers and their students were adapting to a new approach to math instruction while the SB2 group teachers and students continued to use their usual, problem based methods. However in the second year, only the teachers who inherited JUMP group students were new to JUMP, not the students. As implementation fidelity was consistently high from year to year (the large majority of teachers were coded as using their assigned curriculum with high alignment and coder confidence), the results suggest that students in the JUMP group may have experienced latent gains in year 1 that contributed to greater, discernible gains on problem solving in year 2.

All of the primary students initially struggled to make gains on calculation. With its steep item gradient and only a few items that are covered by the early elementary curriculum students would have to advance considerably for detectable effects within a single school year. This may have been especially challenging for the JUMP teachers and students in year 1 as they adapted to the new program. The SB2 group made significantly greater gains in year 1 (for the PP sample but not the HiFi sample) and whereas the SB2 group advanced as much as expected, the JUMP students lost significant ground in year 1 (PP and HiFi samples). But note that the SB2 group lost significantly more ground in the summertime (for the PP sample) and that performance was strongest overall in year 2 when both groups advanced as much as expected (PP and HiFi samples). Thus, an initial buffer against losses for the SB2 students on calculation in year 1 was no longer apparent by the summertime or in year 2.

In general, the two groups performed comparably on math fluency in all time periods. Indeed, the only group difference for math fluency was for the HiFi sample in year 1 when the students in the SB2 group made significantly greater gains than their JUMP peers. But as with calculation, this advantage was not apparent in year 2 (when only the JUMP group made greater than expected gains). For CBC, none of the between group differences were significant (both groups made significant gains in both years, with somewhat greater gains in year 2).

It is interesting that we also found a significant, positive effect of JUMP on broad reading in year 2 (for the PP and the HiFi samples). Given previous research linking affect (such as anxiety and mindset) with math achievement [27, 61, 62], it could be that latent positive effects on math achievement experienced in year 1 bolstered the primary JUMP group’s confidence in their learning, with spillover effects for academic progress more broadly in year 2. This interpretation is in line with Duncan et al’s meta-analysis investigating the contribution of pre-K math and reading skills to academic achievement at the end of elementary school that found that while pre-K math predicts later math achievement (.42) and pre-K reading predicts later reading achievement (.24), pre-K math also predicts later reading (.26) and more strongly than pre-K reading predicts later math (.10) [9]. In other words, early positive experiences with math learning may influence children’s academic self-efficacy more generally. Younger children may be more pliable compared to their older peers in this regard as they may have less entrenched beliefs about their academic abilities.

Finally, unlike the pilot study, the JUMP teachers in the scale-up study did not rate their math teaching experience in the study period more favourably than their comparison peers. But note that all of the groups’ ratings were generally positive.

## General discussion

The present research investigated the effectiveness of JUMP Math, a distinctive approach to math instruction whose key ideas find support in the scientific literature, for improving elementary math achievement. Specifically, we asked if elementary students who received JUMP instruction experienced greater progress in math achievement compared to their same grade peers who received the extant, problem-based approach (the school board or SB students) over the study period. We first carried out a brief pilot study with a relatively small sample of grade 5 (junior) students and their teachers that yielded preliminary evidence of a positive effect of JUMP and suggested that an RCT was feasible. A subsequent, scale-up RCT conducted over two consecutive school years sought to replicate and extend the pilot study findings with a larger sample of junior and primary students and their teachers, and a broader range of outcomes that included measures of students’ ability to apply their mathematical knowledge.

The main findings for the junior students were early positive effects of JUMP instruction on math fluency that persisted over time, when the JUMP group also began to gain some traction on calculation. Their performance on problem solving was comparable to the comparison group in all time periods. That the positive impact of JUMP on computation obtained in two studies conducted with two different school boards strengthens their validity (we did not measure problem solving in the pilot study).

The positive impact on math fluency is an important finding because automatized math facts become stored in long-term memory thus freeing up limited working memory resources that can then be directed towards the conceptual aspects of the math to be learned (such as problem-solving). Previous developmental and neuroscience research has linked the tendency to rely on mathematical procedures (that tax working memory) with poorer math achievement and retrieving mathematical facts from long-term memory with better math achievement. (see e.g. [63–66]) As previously mentioned, significant gains in fluency in year 1 may have helped the JUMP group to make greater overall advances in math in year 2. While performance on standardized measures of math fluency is robustly, positively correlated with performance on applied problems [67], the beneficial impact of JUMP instruction on computation may take longer to manifest on measures of problem solving. Such benefits may have been obscured in the scale up study by the somewhat diluted program delivery in the comparison group teachers in year 2. Note as well, that individual differences in math fluency have been linked to individual differences in the precision of mental number representations and with mental number line linearity, with better fluency related to greater precision and linearity. [68, 69] Taken together, these findings suggest that the benefits of JUMP instruction for the junior students may extend beyond computational skill.

The lack of an SB2 group advantage on problem solving for the junior students is noteworthy because the students in the SB2 group had considerable experience with problem based math instruction coming into the study. The absence of an effect on PSP is particularly striking as the students are used to showing their work and to receiving feedback about the extent to which their answers meet curriculum expectations in their daily lessons. These results are important as they indicate that the positive impact of JUMP instruction on math fluency, can occur without concurrent detriment to progress in problem solving. As mentioned above, improved performance on computation may also confer benefits for problem solving that become discernible in time.

The pattern of results was different for the primary students. Notably, the SB2 group had a significant advantage on measures of problem solving and computation in year 1. This could be because the teachers and students in the SB2 group were using a familiar approach to math instruction while those in the JUMP group were adapting to a new program. The SB2 teachers also continued to receive pedagogical support (as per usual practice). Furthermore, as the SB2 group was using their business-as-usual approach, those teachers also had the benefit of the “community of practice” in their schools while the only teachers using JUMP in the JUMP schools were the grade 2 and grade 5 teachers participating in the study. The stronger performance by the SB2 students in year 1 may thus reflect potential gains with all of these supports in place. But note that the apparent advantages to the SB2 students in year 1 did not persist into year 2.

Indeed, a surprising and important finding was that the JUMP students significantly outperformed their SB2 peers on problem solving in year 2. The benefits to the JUMP group on PSP in particular, were considerable (effect size .26 PP sample, .54 HiFi sample). PSP items are based on problems that have been used in previous years on the regional assessment written annually at the end of grades 3 and 6. Problems are scored according to publicly available guidelines out of 40, with increments of 10 corresponding to a letter grade (e.g. a score between 30 to 40 is in the A range, between 20 and 30 the B range and so on). The observed gains in the JUMP group primary students in year 2 (PP Sample, JUMP 6.2 vs. SB2 3.3 points; HiFi sample, JUMP 7.6 vs. SB2 .6 points) thus correspond to a mean gain of more than half a letter grade, which is a substantial improvement in problem solving.

That the primary students in the JUMP group did not make significantly greater gains on the computational measures compared to their SB2 peers may be because the computational demands in grades 2 and 3 are still sufficiently low that children in the SB2 group could use alternative strategies (such as counting on) to arrive at solutions and perhaps a persistent focus by the students in both groups on problem solving, the approach to instruction the younger children had likely experienced from the start of formal schooling. After temporarily losing ground in progress on calculation by the JUMP group in year 1, the two groups of primary students made similar gains on the computation measures in year 2.

The finding that the primary students lost significant ground in the summertime is consistent with the results from a previous meta-analysis showing substantial summer losses in math. [39] Apart from significantly smaller losses on calculation by the JUMP group in the PP sample, neither group had an overall advantage here. It is not clear why both groups of junior students continued to keep pace with norms even in the summertime since the meta-analysis found that so called summer slide tended to increase with grade level. But note that the research reported predated the implementation of problem-based math instruction, which might have impacted the relation between summer slide in math and grade level.

In sum then, junior students who received JUMP instruction made greater progress on computation, with effects taking hold in the first year of the study, and both groups made similar, concurrent advances in problem solving. In contrast, primary students who received their usual, problem-based math instruction made greater progress on problem-solving and computational measures in the first year. The positive effect of JUMP instruction on primary students took time to manifest, appearing in the second year, and the main impact—on problem solving—occurred without lasting, detriment to progress in computation. The results for both divisions on problem solving are important as problem solving is widely believed to require a deep, conceptual understanding of math and developing good problem solving skills is arguably *the* goal of contemporary math education [22–23]. It is possible that the gains observed in the JUMP students in both divisions on some measures may have a positive influence on progress on the other measures once children, and also their teachers, have fully transitioned to JUMP pedagogy.

### Strengths

Strengths of the present research include random assignment at the school level to curricula, the use of standardized and curriculum-based math outcomes, and the inclusion of computation as well as problem solving measures. Teachers in both groups were able to achieve good implementation fidelity, for those in the JUMP group with relatively little PD, indicating that teachers can take up and implement the JUMP Math program quickly. This, together with the availability of explicit teacher’s guides, smart board lessons and student materials and the teachers’ generally positive experience working with JUMP, suggests that the program that is implementation ready and teacher friendly.

Indeed, a particular strength of the present research was that in both studies we investigated the effectiveness (impact in typical, real-world conditions) as opposed to the efficacy (potential impact in ideal conditions) of the JUMP Math program. Teachers in both groups received PD that was in line with their respective curricula and we did not intervene with program delivery while the study was underway (e.g. to improve implementation fidelity). Children received the same amount and the same schedule of math instruction they normally received prior to study participation. Moreover, the large majority of students were taught by different teachers in the two consecutive school years of the scale-up study, which is typical of what would normally occur in most elementary schools. In addition to standardized measures, we also included math outcome measures that reflect the way that children are expected to approach their daily math work (in the scale up study); they are required to show and often to justify their work and are given credit for appropriate steps taken as well as for producing the correct solution. Moreover, all of the data were collected and coded by teachers with experience working with children who were blind to the study hypotheses and to random assignment to curricula, and who were not otherwise involved in the study. The results of the present research thus provide a good sense of the effects that could arise in the relatively short-term when adopting the JUMP program.

A further strength of the scale-up study was the generally high rate of participation in the teacher observations that provided implementation fidelity data critical to interpreting the student outcomes. That said, we acknowledge that a planned observation of one math class per teacher at mid-year may be limited in its representation of the type of instruction children experienced on a daily basis. We reasoned that more frequent observations might have lowered the participation rate and, more importantly, altered implementation fidelity and the classroom culture more generally, which could influence the study results and their generalizability. However, it remains possible that the teachers in both groups supplemented their assigned approach to math instruction and thus that there may have been some overlap in their math pedagogy.

That the observation participation rate was lower in year 2 compared to year 1 (67–82% year 2 vs. 88–100% year 1), might have been due to differences in teacher motivation. Year 1 teachers consented prior to random assignment to curricula while year 2 teachers joined the study after the randomly assigned curricula were already in place and thus did not share in any excitement that might have surrounded the outcome of random assignment and the launch of a new study. Year 1 teachers in the comparison group also received an additional half-day of math PD delivered by a widely recognized expert in math pedagogy of their choosing. Finally, year 2 teachers may have been somewhat stressed about participating in the study in a high-stakes testing year.

But note that these findings cannot explain the decline in performance in the primary comparison group students from year 1 to year 2 as any effect on teacher motivation in year 2 would have impacted both groups roughly equally. Moreover, the rate of teacher participation in the observations (88% grade 2, 78% grade 3), mean alignment (5.3 grade 2, 4.9 grade 3) and coder confidence (4.2 both years) scores were consistently high, and the incidence of assigned/observed curriculum incongruence (3 classes grade 2, 4 classes grade 3) was similarly low, in the primary division from year to year.

### Limitations

One limitation is that the scale-up study involved schools from a single school board (district). However, the board was fairly large, included both urban and rural schools, and the board results from the most recent regional assessment of academic progress indicated a pattern of findings generally in line with the pattern for the region. The present findings are therefore likely to generalize to other comparable school boards. This notion is supported by the fact that we obtained similar results for the junior students for computation in the pilot and scale up studies that were conducted in different school boards.

Another limitation is that it is possible that the study design may have introduced some bias in the results for the SB2 group. Teachers in the SB2 group received two days of PD, to equate the PD time in the two groups, and year 1 teachers in the scale up study received the additional half day, which is more math focused PD than elementary teachers received prior to the study. Consequently, performance in the SB2 group might have over estimated the gains students would normally have achieved, especially in year 1.

A further limitation is that we were unable to carry out intent-to-treat analysis, which includes all participants randomly assigned to the two groups whether or not they received treatment or later withdrew from the study and is required for a clinical trial. As 2 schools in the pilot study and 1 school in the scale-up study (in the SB1 and SB2 groups, respectively) dropped out after random assignment, we were unable to proceed with recruitment or assessment of the eligible students in those schools. It is therefore not possible to know how data that might have been collected from those students could have influenced the study outcome. On the other hand, the overall completion rate for students over the two years of the study was fairly high.

Note as well that as the large majority of the participating students had different teachers in the two years of the scale-up study, the present study cannot speak to effects that might occur when both teachers and students are familiar with JUMP Math instruction (the number of teachers who participated in both years was too small for meaningful analysis). The possibility of latent gains in year 1 of the scale up study by the JUMP group primary students that became discernible in year 2 suggests that benefits might accrue with greater familiarity. Schools considering taking on the JUMP Math program would therefore do well to commit to a trial period that includes sufficient time when teachers and students have experience working with the program for a fair evaluation.

Finally, the present research also cannot speak to the active ingredients of either the JUMP or SB pedagogy that might have brought about the observed effects. Regarding the positive impact of JUMP, we hypothesize that tailored scaffolding of instruction allows children of all skill levels to experience success at math and that this experience promotes a sense of agency that motivates further efforts that ultimately lead to greater success.

### Conclusions

Randomized controlled trials that evaluate the effectiveness of different approaches to math instruction are essential for helping educators to make informed decisions about how best to invest limited resources while maximizing student gains. In making sense of this research, it may be helpful to keep two considerations in mind. First, as instructional programs represent a constellation of features, that vary from program to program, different programs are likely to impact different outcomes, differentially. In other words, it is unlikely that this research will uncover a so-called silver bullet that brings about large, positive effects across the board, especially in the earliest years of adoption. Second, most evaluation research involves at least one experimental group that receives the novel program and a comparison group that continues to use the methods of instruction that are already in place (i.e. a business-as-usual group), and then comparing the groups’ growth on target outcomes. This means that the groups differ not only in type of instruction, but also in the students’ and teachers’ experience working with them. Effects of the intervention may take time to manifest and may be attenuated while teachers and students are still becoming familiar with it, especially so if the new program differs substantially from previous methods. A new approach to math instruction that yields positive effects on some key measures without concurrent slowing of progress on other key measures in the short-term, when this transition is still underway, may hold considerable promise for more wide-spread, longer term gains. Especially if math achievement scores appear intractable or are declining, the new approach to math instruction may well be a worthy investment.

The relative ease with which teachers were able to take up the JUMP Math program and its positive impact, even in the relatively short-term, on key measures of student math progress point to a viable alternative for math instruction that could be a valuable, evidence-based addition to the teacher’s toolbox. Math instruction programs can impact math learning. Given the role of numeracy in 21^st^ century employability and overall well being, identifying and implementing effective approaches to early math instruction could have far-reaching, positive implications.

## Supporting information

S1 Text Supporting information. (DOCX)

S1 Dataset Pilot study data. (XLSX)

S2 Dataset Scale-Up study data. (XLSX)
