# Supplementary material for: A cluster-randomized controlled trial of the effectiveness of the JUMP Math program of math instruction for improving elementary math achievement
Source: PLoS One. 2019 Oct 30;14(10):e0223049. doi: 10.1371/journal.pone.0223049 (PMC6821143; doi:10.1371/journal.pone.0223049)
Supplement: S1 Text — (DOCX) [file pone.0223049.s001.docx]

Supporting Information

**Introduction**

Consider the results from the most recent international assessment of mathematics achievement in 15-year-olds, the Organization for Economic Co-operation and Development’s Program for International Student Assessment (PISA). Of the 65 participating countries, the United States, United Kingdom, Australia and New Zealand ranked 39^th^, 27^th^, 23^rd^

and ^21st^. Although Germany and Canada fared somewhat better at 16^th^ and 10^th^, their mean scores of 506 and 516, respectively, were considerably behind that of the top five scoring countries - Singapore, Hong-Kong China, Macau, Taiwan and Japan - whose mean scores ranged from 532 to 564. The overall mean for all of the participating countries was 490. [1]. These results should not be surprising. The 2017 National Assessment of Education Progress in the United States (NAEP), for example, found that only 40% and 34% of American 4^th^ and 8^th^ graders, respectively achieve proficiency in mathematics [2]. Entry into the STEM fields is likely to be significantly impacted - only 46% and 31% of American secondary school graduates currently meet benchmarks for college readiness in mathematics and science, respectively [3].

**Program Description**

JUMP stands for Junior Undiscovered Math Prodigies. Table A summarizes the JUMP Math content for each of the five strands of elementary math - number sense, patterning and algebra, measurement, geometry, data management and probability – for each of the participating grades (2, 3, 5 and 6) in the research reported in the present manuscript. The table is followed by detailed descriptions of the JUMP Math finger counting method and an example of a JUMP Math lesson.

The JUMP teacher’s guides provide numerous examples of how to adapt the material for slower or faster moving students. Another example of differentiating instruction according to student needs comes from a lesson on double-digit addition with problems that will require carrying, such as 37 +45. Children who are having difficulty may be asked to complete a set of questions in which they add the numbers in the ones column and then enter the carrying term in the tens column, only. Children who are struggling even at this step may be asked simply to place a check mark beside questions where carrying will be required, without carrying out any addition. In contrast, children who are advancing through the material quickly may be asked to add more than two addends such as 37 + 45 + 64, or addends in the hundreds or thousands such as 377 + 569 or 1038 + 2399. [4]

The student practice and assessment books provide a wealth of review and practice material to alleviate the demands on teachers. However, teachers use their discretion as to how much of the work is completed, based on their students’ progress. Struggling students may benefit from review material or from more practice. If a class is catching on quickly, teachers may decide to skip over some sections of practice material.

Table A. JUMP Math Content By Study Grade

| **a. Number Sense** | | | |
| --- | --- | --- | --- |
| Grade 2 | Grade 3 | Grade 5 | Grade 6 |
| counting forwards, backwards to 100; skip counting to 200; ordering numbers; ways to write a number; reading, writing number words to 20; estimating; more, less , fewer; even , odd; 10s , 1s; equal; addition , subtraction using different strategies; counting coins; addition, subtraction with coins; addition, subtraction of fractions; multiplication, division; using number blocks, charts, number lines, hundreds chart; puzzles; word problems | counting by 2s, 3s, 4s, 5s, 10s, 100s; counting and changing units; ordinal numbers; place values 1s, 10s, 100s; reading, writing number words, writing numbers; rounding to nearest 10s, 100s; estimation; mental math; regrouping; adding, subtracting 2 digits, 3 digits; parts , totals, sums, differences; arrays; doubles; counting money; adding , subtracting bills, coins; converting bills, coins; sets; multiplication, division, remainders; equal parts, parts, wholes; comparing fractions; fractions greater than 1; fractions of length; decimal 10ths; arrangements, combinations; guess and check; using organized lists, charts; puzzles; word problems | place value; writing numbers;  representation with base 10 materials; comparing , ordering numbers; differences of 10, 100, 1 000, 10 000; regrouping; parts, totals; addition, subtraction with 4 digits, 5 digits; strategies for multiplication up to 4 digit by 1 digit , 2 digit by 2 digit, by multiples of 10 up to 10 000; strategies for division; long division up to 4 digits by 1 digit; remainders; mental math; arrays; rounding; rounding decimals; counting, adding , subtracting money; converting different denominations of money; making change; equal parts, parts , wholes; ordering, comparing fractions; mixed, improper fractions; lowest common denominator; decimals; place value and decimals; 10ths, 100ths; adding subtracting, multiplying, dividing decimals; unit rates, proportions; arrangements, combinations; using organized lists, scale diagrams, models; word problems | place value; reading, writing large numbers; representation with base 10 materials; differences from 10 to 10 000; regrouping; adding, subtracting 3 digit numbers; prime, composite numbers, factors; mental math, multiplication to 4 digit by 1 digit , 2 digit by 2 digit, 2 digits by multiples of 10 up to 10 000; long division to 4 digits by 1 digit; 2-digit division; rounding; estimating; sums, differences; counting, converting, adding, subtracting, mental math with different denominations of money; integers; parts, wholes; ordering, adding, subtracting fractions; mixed, improper, equivalent fractions; reducing fractions; lowest common denominator; decimals to 10ths, 100ths; decimals, fractions greater than 1; multiplying decimals by multiples of 10, other whole numbers; dividing decimals by up to 0.0001, unit rates; ratios; equivalent ratios; finding percents; organized lists, circle graphs, other visual representations; word problems |

| **b. Patterns and Algebra** | | | |
| --- | --- | --- | --- |
| Grade 2 | Grade 3 | Grade 5 | Grade 6 |
| finding the pattern core and extending patterns; pattern rules; different ways of showing patterns; reading patterns; predicting terms; growing, shrinking, describing, identifying patterns; finding errors; patterns in 100s charts; problems, puzzles | counting forwards and backwards; increasing and decreasing sequences, attributes; patterns with changing attributes; finding pattern cores; making patterns with squares, extending, identifying a pattern rule; t-tables; patterns involving time; calendars; mixed patterns; describing and creating patterns; 2-D patterns; patterns in the 2, 5, 8 and 9 times tables; patterns with increasing gaps, larger numbers; extending, predicting patterns; equations, adding and subtracting machines; problems, puzzles | counting forwards and backwards; increasing and decreasing sequences; extending patterns using rule; identifying pattern rule; t-tables; repeating, extending, predicting patterns; number lines, lowest common multiples, describing and creating patterns; 2D patterns; number pyramids; patterns in times tables; times tables, circle charts; finding rules for t-tables, patterns; predicting the gap between terms; direct variation; patterns using 1 operation; creating and extending patterns; patterns with large numbers; algebra, variables, equations; problems, puzzles | increasing and decreasing sequences, identifying a pattern rule; extending a pattern, making predictions; t-tables; number lines; lowest common multiples; 2D patterns; describing, creating patterns; finding, applying rules for t-tables, patterns; direct variation; predicting the gap between terms; patterns with larger numbers; advanced patterns; equations, variables; algebraic puzzles; interpreting graphs; representing concepts in t-tables; problems, puzzles |

| **c. Measurement** | | | |
| --- | --- | --- | --- |
| Grade 2 | Grade 3 | Grade 5 | Grade 6 |
| length, width, height; long, short, distance around; units; measuring distance; estimating, comparing length, distance; centimeters, meters; estimating, comparing mass; estimating, measuring, comparing time; clocks, hour, minute; time to hour, minute; quarter past, to; measuring, comparing area; calendars, days, months; thermometers; estimating, measuring capacity; measuring cups; problems, puzzles | estimating, measuring length; rulers; centimeters, meters, kilometers; ordering, assigning units; perimeter; mass; capacity; temperature; analogue, digital clocks; estimating intervals of time, 5 minute intervals; area in square centimeters, half squares, units of area; problems, puzzles | telling time in different ways; second hand; elapsed time; 24 hour clock, temperature; units of length, distance; millimeters, centimeters, decimeters, meters, kilometers, changing units; speed; ordering, assigning appropriate units; math and architecture; problem-solving with measurement; perimeter; circles, irregular polygons; area in square centimeters; area of rectangles, polygons, irregular shapes and polygons; comparing area, perimeter; volume of rectangular prisms; mass; capacity; changing units of measurement; problems, puzzles | mass; milligrams, grams, kilograms; volume of rectangular prisms, elapsed time, subtracting times; 24 hour clock; millimeters, centimeters, decimeters, meters, kilometers; comparing and changing units, using appropriate units of length; applied problems (tall buildings; Welland Canal); exploring, measuring, investigating perimeter; circles and irregular polygons; area in square centimeters, area of rectangles, comparing perimeter, area; area of polygons, irregular shapes, parallelograms, triangles; volume, capacity of rectangular prisms, kilograms and metric tonnes; problems, puzzles |

| **d. Geometry** | | | |
| --- | --- | --- | --- |
| Grade 2 | Grade 3 | Grade 5 | Grade 6 |
| lines, sides, vertices; squares, rectangles, triangles, circles, polygons; matching shapes; symmetry; breaking, creating shapes; cubes, spheres, cylinders, cones, pyramids, prisms; turning 3D shapes, shapes in structures; faces; roll, slide, stack; edges; left, right, above, below; maps; problems, puzzles | sides, vertices, angles; equilateral shapes, quadrilaterals, other polygons; tangrams; congruency; congruency with geoboards, grids; symmetry, lines of symmetry, completing symmetric shapes; comparing shapes, sorting shapes by property; co-ordinate systems, maps; slides, slides on a grid; flips, reflections, flips and slides; turns, rotations, flips, slides, turns; building pyramids, prisms; edges, vertices, faces; pyramid nets, prism nets; sorting 3D shapes; geometry in the world; problems, puzzles | sides, vertices of 2D figures; angles; measuring, constructing angles; angles in triangles, polygons; classifying triangles; constructing triangles, polygons; parallel lines; properties of shapes, special quadrilaterals; congruency; symmetry; comparing, sorting, classifying shapes; co-ordinate systems; slides; co-ordinate maps, grids; reflections, rotations, slides; properties of pyramids, prisms; edges, faces, vertices; nets and faces; sorting 3D shapes; tessellations; patterns with transformations; iso-parametric drawings; geometry in the world; problems, puzzles | sides, vertices of 2D figures; measuring and constructing naming, classifying angles; angles in triangles and polygons; triangles; parallel lines; properties of shapes; special quadrilaterals; diagonal properties of special quadrilaterals; co-ordinate systems and maps; slides, reflections, rotations; transformations on grid and dot paper; properties of pyramids, prisms; edges, faces, vertices; nets, faces; sorting 3D shapes; creating patterns with transformations; iso-parametric drawings; drawing figures, mat plans; orthographic drawings, problems, puzzles |

| **e. Probability and Data Management** | | | |
| --- | --- | --- | --- |
| Grade 2 | Grade 3 | Grade 5 | Grade 6 |
| sorting rules; sorting into groups; sort and graph, pictographs; bar, line graphs; tallies; asking questions about data; surveys, certain , impossible; likely, unlikely; more likely, less likely, equally likely; problems, puzzles | classifying data; Venn diagrams; tallying data; reading tally data from a chart; pictographs, scale; bar graphs, data collection, surveys; interpreting data | classifying data, Venn diagrams; choosing a graph scale; bar graphs; double bar graphs; line graphs; discrete and continuous data; primary and secondary data; samples; surveys; designing, analyzing a survey, designing, analyzing an experiment; mean, median, mode, range; stem and leaf plots; data representation options; outcomes; probability; expectation; games and expectation | Venn diagrams review; bar, double bar, line graphs; stem and leaf plots; making predictions from graphs; continuous, discrete data; scatterplots; mean, median, mode, range; choosing, interpreting graphs; primary, secondary data; samples, surveys; sample bias; designing, displaying, analyzing surveys and experiments; outcomes; probability; expectation; games and expectation; tree diagrams; counting combinations; compound events; puzzles, problems |

**JUMP Math Finger Counting Method**

Students at all ages are encouraged to use the JUMP Math finger counting method as a computational aid. Children can use this method to assist with counting by 1’s, skip counting (counting by units greater than 1), addition, subtraction and multiplication. It is recommended to begin with skip counting by 2’s and 5’s. Once children are accustomed to using their fingers in this way, the method can be adapted to assist with operations. The procedures for skip counting, addition, subtraction and multiplication are shown below.

Skip Counting


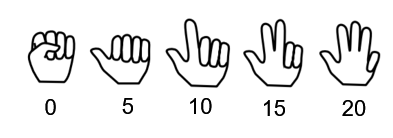

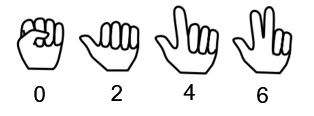


Skip counting by 2’s Skip counting by 5’s

Show the child a closed fist and say aloud “0”. Raise your thumb, then each finger in turn while saying the multiples of the number you are counting by. For example, for skip counting by 2’s, say “2, 4, 6” (see image above). To skip count by 2’s higher than 10, continue counting with the fingers on the other hand as needed. Have the child practice these procedures for 5-10 minutes each day until they can execute them with ease. Begin with skip counting by 2’s and with shorter counting sequences, extending the sequence length as the child becomes more proficient. The same procedure can be adapted and used for skip counting by 5’s (see above) or any other number.

Addition


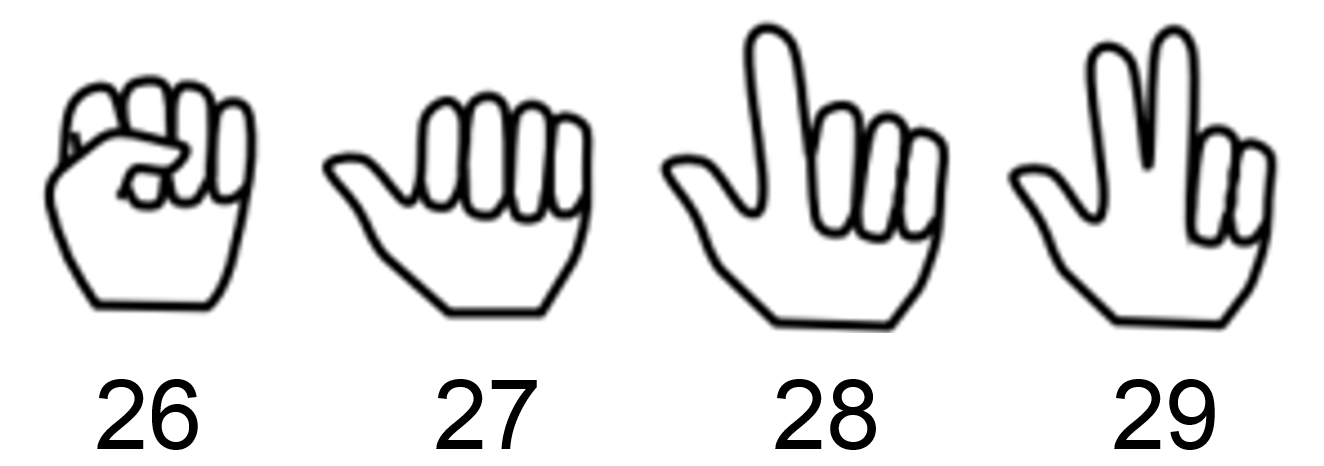


26 + 3 = 29

where you

stopped counting

number you started with

number of fingers raised
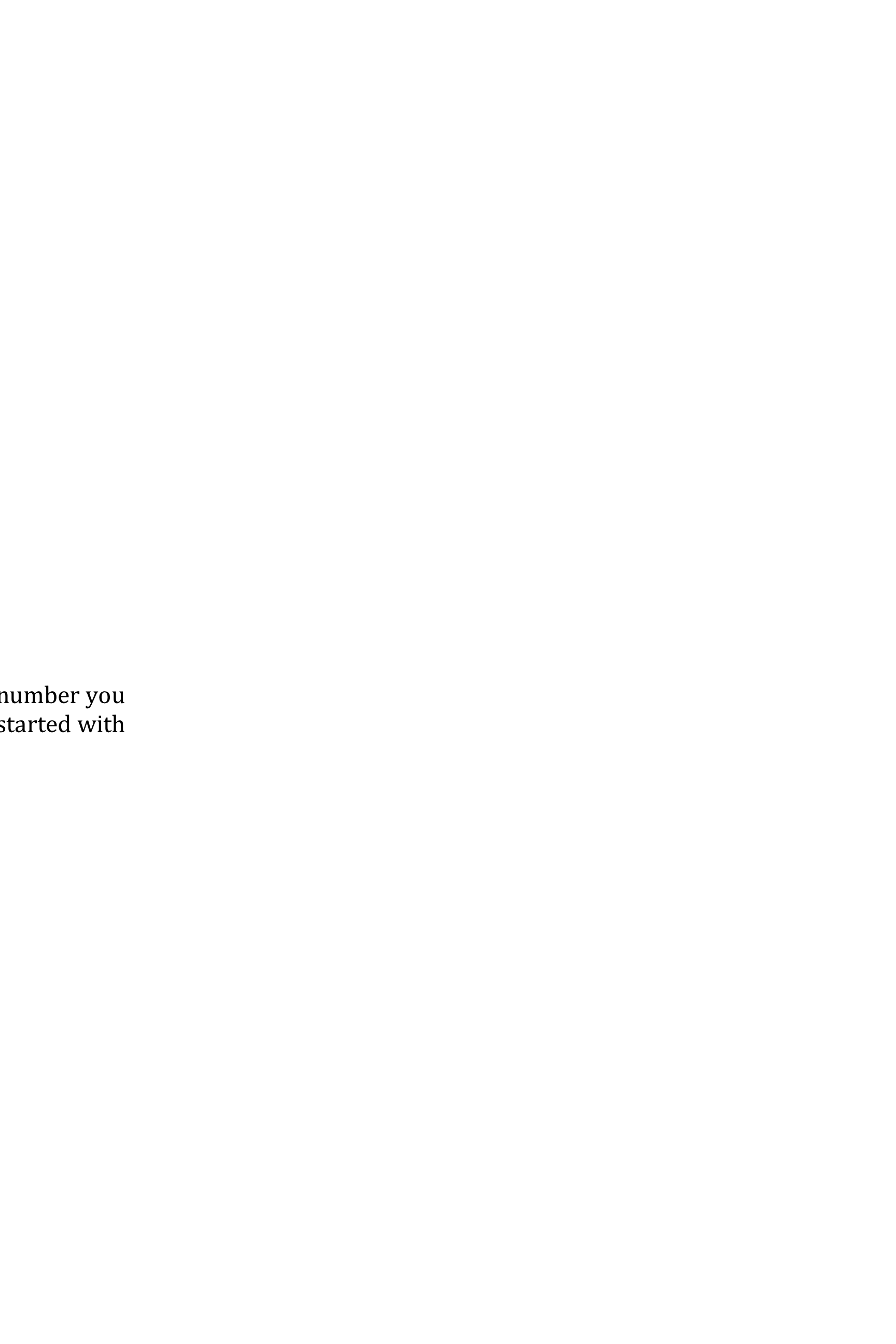


Extend an open hand and “catch” the largest addend into your closed fist saying the number aloud. Then raise your thumb and each finger in turn as you continue to count aloud by 1’s until the number of fingers raised matches the smaller addend. For example for 26 + 3, “catch” the number 26 into your closed fist and then raise your thumb and each finger in turn as you say “27, 28, 29”. Explain that the number of fingers raised is the number you added and that the last number you say is the answer to the addition question (the sum). To add numbers greater than 5, continue counting with the fingers on your other hand. To practice, “throw” the largest addend to a single child (or to a group of children) and then tell them the number to add. Ask them to give you the answer.

When children are accustomed to the addition procedure, you can connect the process to a written addition sentence. For example, write “26 + 3 = 29”. Explain that the first number shows the number you started with, the next number is how many fingers were raised when you stopped counting and the last number is the answer to the addition question.

To increase enjoyment and motivation, increase the magnitude of the larger addend while keeping the other addend relatively small e.g. 450 + 5 or 1262 + 8.

Subtraction


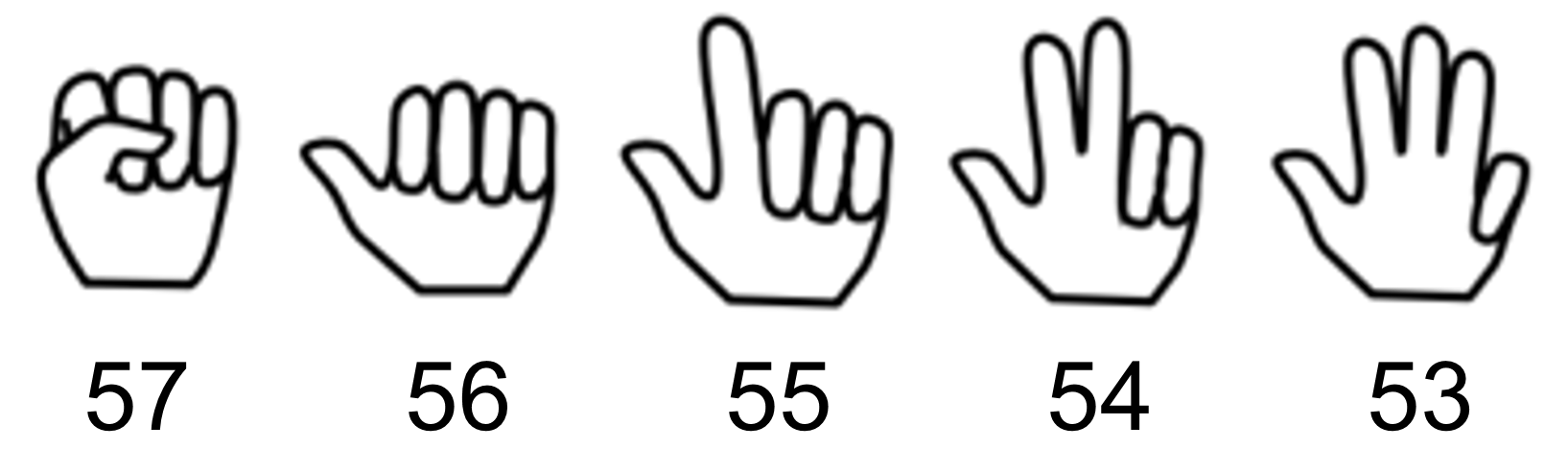


57 - 4 = 53

number you started with

where you stopped counting

number of fingers raised
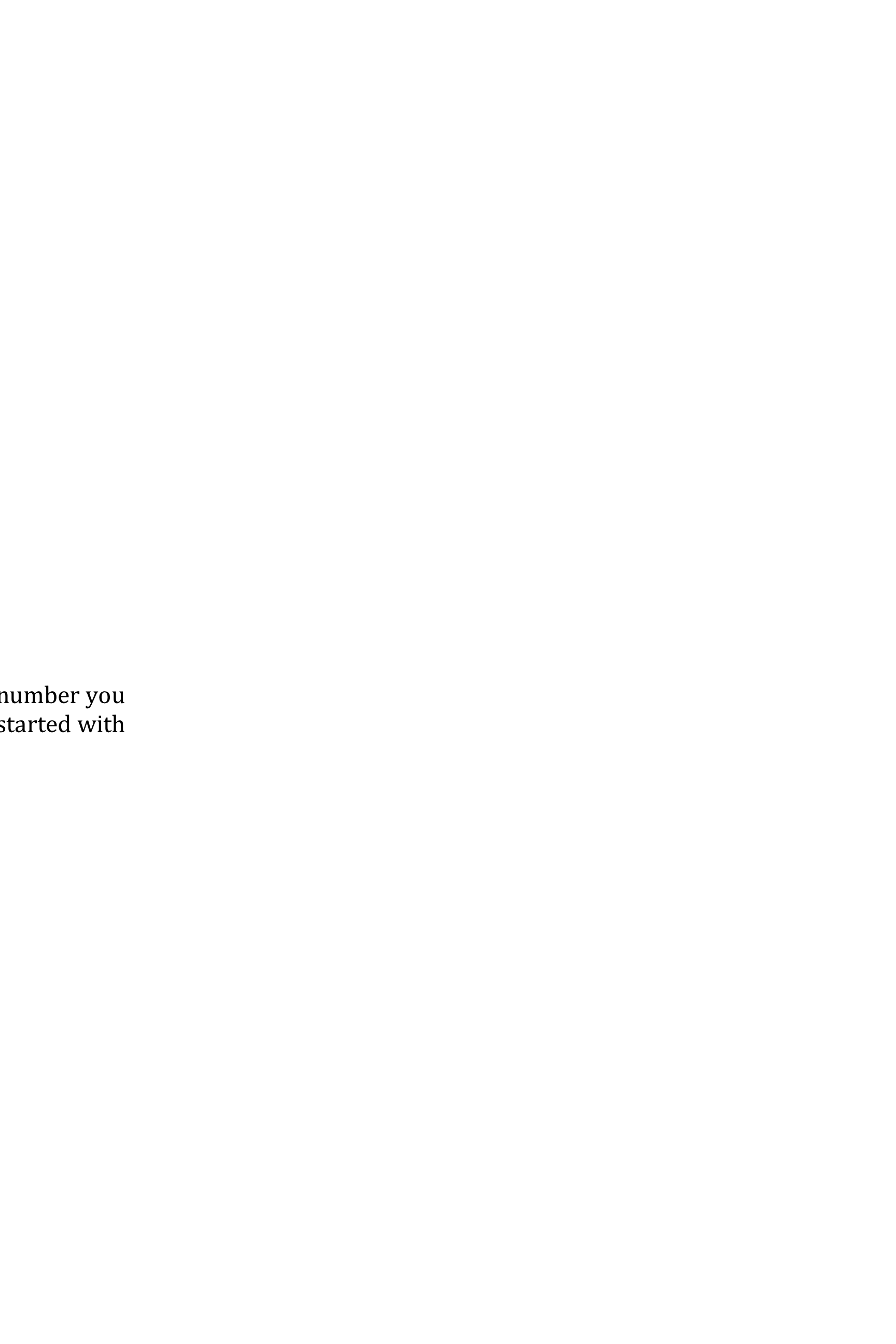


As for addition, but begin by catching the minuend (the number you are starting with) in your enclosed fist and saying the number aloud. Then raise your thumb and each finger in turn as you count down by 1’s until the number of raised fingers matches the subtrahend (the number you are taking away). The last number you say is the answer to the subtraction question (the difference). For example, for 57- 4, “catch” the number 57 into your closed fist and then raise your thumb and each finger in turn as you say “56, 55, 54, 53”. Explain that the number of fingers raised is the number you are taking away and that last number you say is the answer to the subtraction question. To subtract numbers larger than 5, continue counting down with the fingers on your other hand. To practice, throw the minuend to a child (or to a group of children), then tell them the number to take away. Ask them for the answer.

To connect the process to a written subtraction sentence, write “57 – 4 = 53”. Explain that the first number shows the number you started with, the next number shows the number of fingers raised when you stopped counting down and the last number is the answer to the subtraction question.

To increase enjoyment and motivation, increase the magnitude of the minuend while keeping the subtrahend relatively small e.g. 360 - 6 or 1100 - 8.

Multiplication


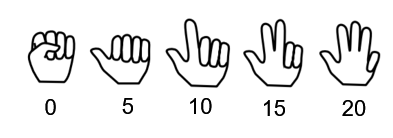


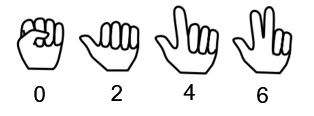


2 x 3 = 6 4 x 5 = 20

number of

fingers counted

where you

stop counting

where you

stop counting

number of

fingers counted

what each

finger represents
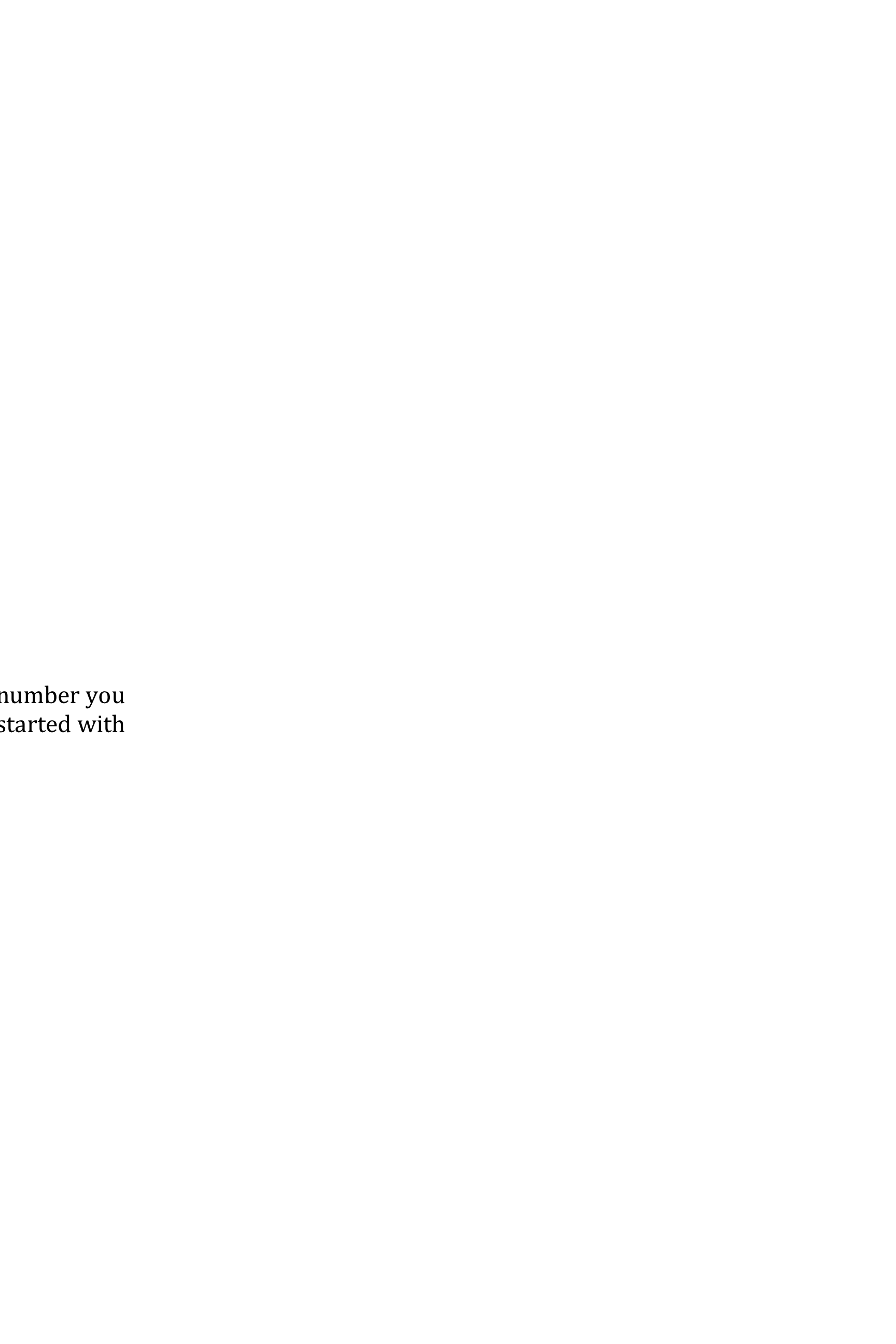


what each

finger represents
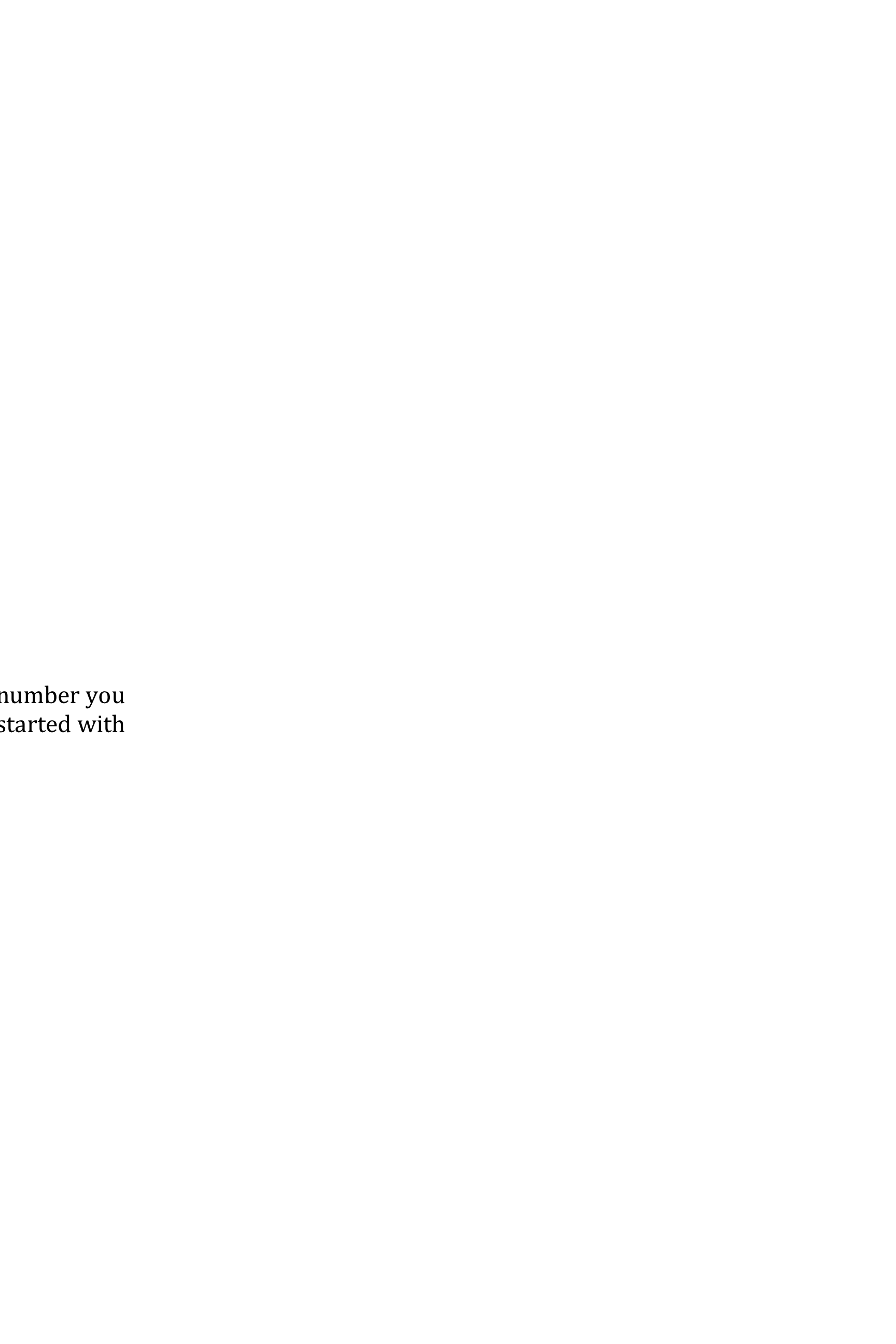


Once children have mastered skip counting by 2’s and 5’s, you can extend the method to teach the 2 and 5 times multiplication facts. For example, to teach multiplying by 2’s, begin with the procedure for skip counting by 2’s above. Then explain that each finger indicates that you counted 2 things and that the number of fingers raised when you stop counting shows how many groups of 2 you counted. Have the child practice with different multiples of 2. For multiples greater than 5, continue counting with the fingers on your other hand. Each time the child stops counting, check that they know how many things each finger means and how many groups they counted.

Connect this process to a written multiplication sentence. For example, write “3 x 2 = 6” and explain that the first number shows the number of fingers or groups you counted and the second number is the number of things in each group (e.g. that there are 2 things in each group). Next, explain that the last number you say is the answer to the multiplication question. Have the child practice, beginning with smaller multiples and continuing until they can multiply up to 2 x 10 in this way with ease. Repeat the procedure to teach multiplying by 5’s.

The procedures for teaching multiplication by 2’s and 5’s are part of a more comprehensive system of teaching the multiplication facts up to 10 x 10 using number patterns, that was developed to help children commit their multiplication facts to memory, and is part of the JUMP Math program [5].

**Example of a JUMP Math Lesson**

Here we describe the steps for a grade 5 lesson on long division that demonstrates JUMP pedagogy including how teachers use direct instruction to guide students to discover the steps of the standard algorithm, to understand the underlying concepts and to become proficient at using the algorithm [6]. In this lesson, the teacher expresses a long division problem in terms of sharing coins (10 cent pieces and 1 cent pieces) equally amongst friends.

**Pedagogical Steps for Grade 5 Lesson on Long Division**

Step 1: Tell the students that one way to think about the following notation:

3 $\text{)72}$ , is that it indicates 3 friends wanting to share 72 cents, equally.

Step 2: Ask them to draw a picture showing how they would first divide the 10-cent pieces amongst the friends. If they use a box for each friend and an X for each 10-cent piece, their drawing might appear as follows:

XX

XX

XX

X

Step 3: Ask the students to explain the picture; there are 3 friends (boxes), each friend gets 2 10-cent pieces and there is 1 10-cent piece left over.

Step 4: Show them how someone writing out the first few steps of long division might show the same information:

2

3 $\text{)72}$

- 6

1

Step 5: Ask students to determine what each step in the algorithm means by identifying where they see each number in their picture (above). Students readily determine that:

2 number of 10-cent pieces each friend receives

3 $\text{)72}$

- 6 number of 10-cent pieces given away so far

1 number of 10-cent pieces left over

Step 6: Ask the students to complete their original picture, showing how much money is left to be divided. If they use an O for the 1-cent pieces, their drawing might look like this:

XX

XX

XX

X 0 0

Step 7: Invite 3 students to the front of the class and demonstrate sharing the remaining coins – give 2 students 1 1-cent piece each and 1 student the remaining 1 10-cent piece.

Students will readily protest that this is an unfair distribution of the money.

Step 8: Ask them how you can solve this problem, so the money is distributed fairly. Students will readily suggest that trading in the 1 10-cent piece for 10 1-cent pieces, will yield a total of 12 1-cent pieces that could then be divided amongst the friends.

Step 8: Tell students that exchanging the 10-cent piece as 1-cent pieces is called regrouping and that in the long division procedure it is showing by “bringing the number down”. Show them by continuing on with the algorithm:

2

3 $\text{)72}$

- 6

12 the number you are “bringing down”

Step 9: Explain that when you bring down the number in the 1’s column, you are changing the number in the 10’s column into the smaller unit (1’s) and that combining all of the smaller units gives you 12 1-cent pieces.

Step 10: As them to show in their original picture how they would distribute the 12 1-cent pieces:

XXOOOO

XXOOOO

XXOOOO

Step 10: Now ask them to connect the numbers in their picture to the remaining steps in the algorithm:

24 each friend received 4 1-cent pieces (24 cents altogether)

3 $\text{)72}$

- 6

12

- 12 12 1-cent pieces were given out altogether

0 no 1-cent pieces left over

Note that at each step of the process you should give students practice questions to ensure they have mastered each step. If students are struggling at a given step, give more practice questions demonstrating only that step until the students become proficient at that step of the learning process.

**Pilot Study**

**Participants**

Three percent of elementary students in the participating school board were born outside Canada, 2% had a non-English first language and 1% were English language learners (the corresponding provincial values were 12%, 22% and 9%, respectively). Thirty-four percent required special education (provincial value 20%). The percentage of junior elementary students who reached expectations on the annual, provincial math assessment in the year preceding the study was 54.6% (sd = 18.35) in the participating JUMP schools, and 56.3% (sd = 19.65) in the SB1 schools (provincial mean 56%)(see note [7]).

School socioeconomic data were based on a needs index created by the school board; schools were ranked according to their needs on five factors - poverty, family/community demographics, mobility, cultural/linguistic diversity and readiness to learn (based on data from publicly available databases such as Statistics Canada) – and the board then calculated the mean of the five rank scores for each school. Higher mean rankings indicate schools with greater needs. The range of the mean ranks was 9.8 - 58.6 for all of the elementary schools in the school board and 23.4 - 58.2 for the participating schools. The mean rank for the participating schools was: 39.2 (sd = 9.96) JUMP and 35.8 (sd = 8.24) SB1.

Teachers in the JUMP group had mean of 10.1 years (range .33 to 23 years) of teaching experience, those in the SB1 group had a mean of 13.5 years (range 3 to 30 years) of teaching experience. The percentage who last studied math in high school was 61% and 45% for the JUMP and SB1 schools, respectively. The rest had some university level math. (see note [8] [9-11]

**Class Composition**

It is commonplace in Ontario elementary public schools to have both “straight” grade classes comprising children in the same grade who are taught by one teacher, as well as “split” grade classes comprising children in two adjacent grades, all taught by one teacher. For example, a teacher could have a class with some grade 2 and some grade 3 students (a 2/3 split) or some grade 3 and some grade 4 students (a 3/4 split). A student in grade 3 could therefore be in a straight grade 3, in a low split (grade 2/3) or in a high split (grade 3/4). Split grades allow schools to maximize classroom capacity (established by policy makers, for each grade) while minimizing the number of teachers. The proportion of straight and split classes can vary widely from grade to grade within a school, and also from school to school, according to the student population. Principals work with teachers in the spring to determine the composition of classes for the following school year. Teacher and student assignment to the different types of grades is based on a number of variables including the number of students in each grade, gender, overall functioning (academic, social, emotional), student dynamics, teacher-student suitability and, to the extent possible, teacher willingness to teach a particular type of grade. The distribution of the different types of grades in the two studies is shown in Table B (see note [8]) [9-11].

Table B. Distribution of Low Split, Straight and High Split Classes in the Two Studies.

|  | **JUMP** | | | **SB1 (pilot) or SB2 (scale-up RCT)** | | |
| --- | --- | --- | --- | --- | --- | --- |
|  | Low Split  (%) | Straight  (%) | High Split  (%) | Low Split  (%) | Straight  (%) | High Split  (%) |
|  | | | | | | |
| **Pilot Study** |  | | | | | |
| Grade 5 | 6  (33) | 6  (33) | 6  (33) | 3  (27) | 2  (18) | 6  (11) |
|  | | | | | | |
| **Scale-Up RCT** |  | | | | | |
| Grade 2 | 7  (23) | 17  (54) | 7  (23) | 5  (19) | 10  (39) | 11  (42) |
| Grade 3 | 10  (29) | 16  (47) | 8  (24) | 5  (18) | 14  (52) | 8  3(0) |
| Grade 5 | 6  (30) | 13  (48) | 8  (22) | 9  (41) | 9  (41) | 4  (18) |
| Grade 6 | 3  (12) | 16  (64) | 6  (24) | 8  (38) | 10  (48) | 3  (14) |

A low split has students in the target grade and in the lower grade e.g. grades 2 and 1.

A high split has students in the target grade and the higher grade e.g. grades 2 and 3.

In a straight grade all students in the class are in the target grade. SB1/2 (school board 1/2) denotes the comparison groups in the participating school boards.

The curriculum for a given grade is the same regardless of the type of grade (straight, low or high split). In a split grade class, some lessons are taught to the class as a whole with different work assigned and/or different expectations for the work according to the student’s grade level. For other subjects such as math, teachers gather together and teach a lesson to all of the students in a single grade, while the students in the other grade complete assigned work either at their desks or in another area of classroom.

In both studies, participating teachers used grade appropriate material from their assigned method of instruction (JUMP or SB1/SB2) with all of the students in the class i.e. including those students in the grade that was not participating in the study. For example, a teacher in the JUMP group teaching a 1/2 split would use grade 1 JUMP materials for the grade 1’s and grade 2 JUMP materials for the grade 2’s, even though only the grade 2’s were participating in the study.

The number of classes in each grade type in the two groups in the pilot study was too small for meaningful analysis of the effect of type of grade on growth in math achievement. See the beginning of the results section for the scale up study (in this document) for our findings regarding this issue with the larger sample.

**Measures and Procedures**

Math Achievement. For math fluency, students complete as many single-digit addition, subtraction and multiplication questions as possible within a 3-minute time limit. For calculation, questions ranging from single digit addition to functions and matrices are arranged in order of difficulty and students complete as many questions as possible with no time limit. Quantitative concepts probes knowledge of concepts such as time, money and operations and students are tested to a criterial number of errors.

Additional Measures. For letter-word identification, participants are presented with displays of letters and words and asked to identify specific letters, find particular words, read lists of words and so on. Verbal and non-verbal IQ were measured with the verbal knowledge plus riddles, and the matrices subscales (respectively) from the Kaufman Brief Intelligence Test (KBIT [12]) and working memory with the backwards version of the non-word letter span test adapted from the WISC-III; a string of letters (that do not form a word) is read aloud and students repeat the list reversing the order of presentation. [13] Table C shows the schedule of administration of the various measures in the two studies.

| Measure | Pilot Study | |  | Scale-Up Study | | | | |  |
| --- | --- | --- | --- | --- | --- | --- | --- | --- | --- |
|  |  | |  | Year 1 | | Year 2 | | |  |
|  | Fall  (Pre-) | Spring  (Post-) |  | Fall  (T1) | Spring  (T2) | | Fall  (T3) | Spring  (T4) | Test  Reliability |
| **Math Achievement** |  |  |  |  |  | |  |  |  |
| Math Fluency^a^ | ✓ | ✓ |  | ✓ | ✓ | | ✓ | ✓ | 0.90^b^ |
| Calculation^a^ | ✓ | ✓ |  | ✓ | ✓ | | ✓ | ✓ | 0.86^b^ |
| Quantitative Concepts^a^ | ✓ | ✓ |  | - | - | | - | - | 0.91^b^ |
| Applied Problems^a^ | - | - |  | ✓ | ✓ | | ✓ | ✓ | 0.93^b^ |
| Broad Math Cluster^a^ | - | - |  | ✓ | ✓ | | ✓ | ✓ | 0.95^b^ |
| Problem-Solving Process |  |  |  | ✓ | ✓ | | ✓ | ✓ | - |
| Curriculum Based Computation |  |  |  | ✓ | ✓ | | ✓ | ✓ | 0.95-0.98^c^ |
|  |  |  |  |  |  | |  |  |  |
| **Reading Achievement** |  |  |  |  |  | |  |  |  |
| Letter-Word Identification^a^ | ✓ | ✓ |  | ✓ | ✓ | | ✓ | ✓ | 0.94^b^ |
| Reading Fluency^a^ | - | - |  | ✓ | ✓ | | ✓ | ✓ | 0.90^b^ |
| Passage Comprehension^a^ | - | - |  | ✓ | ✓ | | ✓ | ✓ | 0.88^b^ |
| Broad Reading Cluster^a^ |  |  |  | ✓ | ✓ | | ✓ | ✓ | 0.94^b^ |
|  |  |  |  |  |  | |  |  |  |
| **Additional Measures** |  |  |  |  |  | |  |  |  |
| IQ verbal/non-verbal | ✓ |  |  | ✓ |  | |  |  | 0.90/0.88^d^ |
| Working Memory | ✓ |  |  | ✓ |  | |  |  | 0.87^e^ |
| Processing Speed | - | - |  | ✓ |  | |  |  | 0.84-0.92^f^ |

Table C. Schedule of Administration of the Student Measures in the Two Studies

^a^From the Woodcock-Johnson III Achievement Battery [14]. Broad math cluster scores are based on the math fluency, calculation and applied problems. Broad reading cluster scores are based on letter-word identification, reading fluency and passage comprehension. ^b^From the Woodcock-Johnson III Technical Abstract [15]; ^c^From Monitoring Basic Skills Progress: Basic Math Computation [16]; ^d^From Kaufman Brief Intelligence Test [12]; ^e^From WISC-III as a Process Instrument [13]; ^f^From Wolf & Denckla [17].

**Results**

In both studies, results for the main math and reading outcomes from the WJ-III are based on standard scores. Previous research has shown that normative data for the WJ-III obtained from American students are suitable for gauging achievement in Canadian students. [18] Indeed, the WJ-III battery is routinely used by Canadian school psychologists for assessing individual academic progress. We carried out the same analyses for the main math outcome measures using standard scores as well as *W* scores, scaled scores that are also produced by the WJ-III scoring software and widely reported in the literature. [19] The pattern of results for the two types of scores was highly similar. We report the results for standard scores, as they are easier to interpret and hence may be more accessible to a broad audience. Table D shows a summary of the results in the pilot study.

Table D. Mean Estimates of Change in the Pilot Study

|  | **JUMP**  **Estimate (95%CL)**  **p-value** | **SB1**  **Estimate (95%CL)**  **p-value** | **JUMP minus SB1**  **Estimate (95%CL)**  **p-value** | **Effect Size** |
| --- | --- | --- | --- | --- |
|  |  |  |  |  |
| **Math Fluency** | | | | |
| Pre-Post  Change | 2.3 (.99;3.58)  .001 | -0.3 (-1.99;1.39)  .72 | 2.6 (0.46;4.71)  **0.001** | **.70** |
| **Calculation** | | | | |
| Pre-Post  Change | -.4 (-2.01;1.24)  0.63 | -2.5 (-4.56;-0.35)  .02 | 2.1 (-.58;4.73)  0.6 | .43 |
| **Quantitative Concepts** | | | | |
| Pre-Post  Change | 3.6 (1.85;5.37)  .0003 | 2.1 (-.22;4.36)  .07 | 1.5 (-1.36;4.43)  >0.9 | .30 |
| **Reading** | | | | |
| Pre-Post  Change | .9 (.11;1.76)  .03 | .6 (-.46;1.61)  0.27 | .4 (-.97;1.68)  0.6 | .15 |

Reading is based on the Letter-Word Identification scale, all scales from the Woodcock-Johnson III achievement battery. [14] Results are based on standard scores. A positive effect size value indicates better performance in the JUMP group, a negative value better performance in the SB2 group. An effect size of .25 has traditionally been considered educationally meaningful [20]. A recent meta-analysis of Education intervention research found that effect sizes are rarely as large as .30 and hence that effect sizes of .50 would be considered very large indeed [21].

**Scale-Up Study**

**Implementation Fidelity**

We also conducted spot checks; a member of our research team familiar with the coding scheme selected 25 files (14% of the total number of videotapes), roughly equally from the 3 coders, and coded the type of curriculum observed. These tapes included all 15 cases (9 JUMP, 6 SB2) where coder confidence was <4 out of 5 (the remaining 10 tapes were randomly selected). The research team member and the coder agreed about the type of curriculum observed for all but 1 of the videotapes (96%).

Table E. Mean Percentage of Time Spent in Teacher Roles and Student Configurations

|  | Primary Division | | | | Junior Division | | | |
| --- | --- | --- | --- | --- | --- | --- | --- | --- |
|  | Grade 2 | | Grade 3 | | Grade 5 | | Grade 6 | |
|  | **JUMP** | SB2 | **JUMP** | SB2 | **JUMP** | SB2 | **JUMP** | SB2 |
| **Teacher Role** |  |  |  |  |  |  |  |  |
| Direct Instruction | **43.9** | 16.7 | **50.7** | 12.5 | **48** | 10.3 | **36.3** | 26.5 |
| Facilitating | **8.6** | 28.8 | **7.7** | 32.8 | **5.1** | 32.6 | **13** | 19.8 |
| Circulating | **32.8** | 39.2 | **28.9** | 35.8 | **39.1** | 40.4 | **33.3** | 35.6 |
| Review | **6.5** | 5.8 | **5.0** | 8.1 | **4.1** | 6 | **4.1** | 1.3 |
| Organizing | **2.9** | 6.0 | **2.3** | 7.1 | **0.6** | 3.5 | **1.5** | 3.6 |
| Available | **5.1** | 3.4 | **5.0** | 3.7 | **3** | 7 | **11.8** | 13.2 |
| Distracted | **0.2** | 0.1 | **0.4** | 0.0 | **0.1** | 0.2 | **0** | 0 |
|  | | | | | | | | |
| **Student Configuration** |  |  |  |  |  |  |  |  |
| Individual | **28.1** | 11.7 | **26.1** | 19.6 | **32** | 6 | **36** | 29 |
| Small Groups/Pairs | **11.5** | 35.6 | **11.1** | 29.2 | **9** | 41 | **12** | 21 |
| Whole Class | **60.4** | 52.7 | **62.8** | 51.2 | **59** | 53 | **52** | 50 |

**Measures**

Math Achievement. Applied problems from the WJ-III achievement battery [14] taps children’s ability to apply their mathematical knowledge to solve word problems involving concepts such as time, distance, money and geometry. Children are tested to a criterial number of errors. For curriculum-based computation (CBC), children complete as many computation questions as possible within a number of minutes that is equal to their grade (e.g. 2 minutes for grade 2). [16]

Problem-Solving Process (PSP) Development and Scoring

For each test, we randomly selected one grade appropriate question corresponding to each of the five elementary math strands (number sense, patterning and algebra, data management and probability, measurement and geometry) from the item pool, which was publicly available at the time. We adapted the questions by reframing the problem context (e.g. from eggs in a basket to stickers in a cup) as well as the magnitudes involved (e.g. replacing the number 245 with 265), while keeping the problem structure (and therefore the mathematical demands) the same. As the original items were created for grades 3 and 6, we further adapted them for grade 2 and 5 by ensuring that the quantities were in the appropriate range (e.g. in the ten’s for grade 2 rather than in the 100’s as in grade 3). Furthermore, to avoid confounding the younger children’s writing and math skills, for the primary students only, the tester read the problem-aloud, children responded orally, and the tester noted children’s responses on the test in writing. Testers recorded what the children said, without any prompting or further probing.

PSP data from all four data collection time points were scored after T4, by teachers who had been part of the data collection team (but did not participate in the study). The primary and junior responses were scored by two different groups of teachers. Tests for a given time point (e.g. T2) were scored to completion before moving on to another time point, until all four time points had been scored. Tests were randomly assigned to each scorer with the caveat that no one scored a test they had administered. For each time point, the first 20% of each scorer’s assignment was also randomly assigned to another teacher in the same scoring group for second scoring, for purposes of calculating inter-rater reliability. As increments of 10 points on the final test score correspond to the levels of math skill that are reported for the regional assessment (level 1 indicates a score between 0 to 10; level 2, 11 to 20; level 3, 21 to 30 and level 4, 31 to 40), inter-rater reliability was based on agreement on level of math progress (1, 2, 3, or 4). Mean inter-rater reliability (across the 4 waves of data collection) was 81% for the primary division students and 89% for junior division students. The higher value for the junior students likely because student’s responses were more fleshed out and somewhat less variable compared to the primary students. We also calculated inter-rater reliability according to agreement on whether or not test scores met regional expectations i.e. level 3 or higher. Mean inter-rater reliability based on this approach was 92% and 97% for the primary and junior divisions, respectively. As inter-rater reliability was high, we only used the data from the original scorer in our analyses.

Additional measures. For reading fluency, participants read a sentence and must determine if its meaning is true or false (e.g. a cow is an animal). They complete as many items as possible within a 3-minute limit. For passage comprehension, students point to pictures that correspond to the meaning of words, phrases and passages the experimenter reads aloud. Students are tested to a criterial number of errors. [14] For Rapid Automatized Naming, participants are presented with a 5 by 10 array of either letters or numbers and are required to name each item, from left to right and top to bottom, as quickly as possible. The experimenter records the time to name all 50 items. [17]

**Results**

Preliminary analyses of the scale-up study data indicated no significant interactions between group differences in growth in math achievement and type of grade (low split, straight, high split) on any of the main outcome measures. Thus, the data were collapsed across type of grade in our analyses of the impact of curriculum on growth in math (and reading) achievement.

Mean estimates of change for the two groups, in the three time periods, are shown for the Junior and Primary per protocol samples in Tables F and G and for the Primary high fidelity sample in Table H. As in the pilot study, effect sizes were derived from estimates from the hierarchical repeated measures models.  We calculated standard deviation for the change in each time period by multiplying the standard error of the estimate of change for each condition by the square root of its degrees of freedom.  Effect size is the change in the JUMP group minus the change in the SB2 group, divided by the pooled standard deviation. Hence, a positive value indicates better performance in the JUMP group, a negative value better performance in the SB2 group.

Table F. Estimates of Mean Change in the Scale-Up Study: Junior (grade 5/6) Per Protocol Sample

|  | **JUMP**  **Estimate (95%CL)**  **p-value** | **SB2**  **Estimate (95%CL)**  **p-value** | **JUMP minus SB2**  **Estimate (95%CL)**  **p-value** | **Effect Size** |
| --- | --- | --- | --- | --- |
|  |  |  |  |  |
| **Broad Math** | | | | |
| Year 1 | 1.2 (0.2;2.2)  0.01 | 1.0 (0.0;2.0)  0.06 | 0.2 (-1.2;1.6)  0.7 | 0.04 |
| Summer | 0.6 (-0.4;1.5)  0.2 | -0.3 (-1.4;0.8)  0.6 | 0.9 (-0.5;2.3)  0.2 | 0.14 |
| Year 2 | 1.7 (0.7;2.7)  0.001 | 0.2 (-0.9;1.3)  0.7 | 1.5 (0.1;2.9)  **0.04** | **0.22** |
| **Broad Read** | | | | |
| Year 1 | 3.3 (2.5;4.1)  <.0001 | 3.6 (2.8;4.4)  <.0001 | -0.4 (-1.4;0.7)  0.5 | -0.06 |
| Summer | 1.1 (0.5;1.8)  0.001 | 1.1 (0.4;1.9)  0.004 | 0.0 (-1.0;1.0)  >0.9 | -0.01 |
| Year 2 | 1.3 (0.6;2.1)  0.0005 | 0.7 (-0.1;1.5)  0.07 | 0.6 (-0.5;1.6)  0.3 | 0.11 |
| **Applied Problems** | | | | |
| Year 1 | 1.7 (0.8;2.6)  0.0002 | 2.1 (1.1;3.0)  <.0001 | -0.3 (-1.6;0.9)  0.6 | -0.05 |
| Summer | 0.7 (-0.2;1.5)  0.1 | -0.4 (-1.4;0.6)  0.5 | 1.0 (-0.3;2.4)  0.1 | 0.15 |
| Year 2 | 0.6 (-0.4;1.5)  0.2 | -0.5 (-1.5;0.5)  0.3 | 1.1 (-0.3;2.4)  0.1 | 0.14 |
| **Calculation** | | | | |
| Year 1 | -0.1 (-1.6;1.3)  0.9 | -0.2 (-1.8;1.3)  0.8 | 0.1 (-1.9;2.1)  >0.9 | 0.01 |
| Summer | 0.3 (-1.1;1.7)  0.7 | 0.1 (-1.4;1.7)  0.9 | 0.2 (-1.9;2.3)  0.9 | 0.02 |
| Year 2 | 2.1 (0.5;3.6)  0.008 | 0.2 (-1.4;1.9)  0.8 | 1.9 (-0.3;4.0)  0.09 | 0.17 |
| **Math Fluency** | | | | |
| Year 1 | 2.2 (1.2;3.2)  <.0001 | 0.1 (-1.0;1.1)  0.9 | 2.2 (0.8;3.5)  **0.002** | **0.31** |
| Summer | 0.9 (0.0;1.8)  0.05 | 0.4 (-0.7;1.4)  0.5 | 0.5 (-0.8;1.9)  0.4 | 0.08 |
| Year 2 | 3.0 (2.0;4.1)  <.0001 | 1.6 (0.5;2.8)  0.006 | 1.4 (-0.1;2.9)  0.07 | 0.15 |
| **Problem-Solving Process** | | | | |
| Year 1 | 5.4 (4.2;6.6)  <.0001 | 6.3 (5.0;7.5)  <.0001 | -0.9 (-2.6;0.8)  0.3 | -0.14 |
| Year 2 | 4.5 (3.2;5.8)  <.0001 | 3.4 (2.0;4.7)  <.0001 | 1.1 (-0.7;2.9)  0.2 | 0.14 |
| **Curriculum Based Computation** | | | | |
| Year 1 | 4.3 (2.4;6.3)  <.0001 | 2.7 (0.7;4.6)  0.009 | 1.7 (-1.0;4.4)  0.2 | 0.17 |
| Year 2 | 2.1 (0.1;4.1)  0.04 | 1.5 (-0.6;3.6)  0.2 | 0.6 (-2.1;3.4)  0.7 | 0.06 |

Broad math comprises applied problems, calculation and math fluency and results for these measures and for broad reading are based on standard scores. Problem-solving process and curriculum based computation results are based on raw scores and percent correct, respectively. A positive effect size value indicates better performance in the JUMP group, a negative value better performance in the SB2 group. An effect size of .25 has traditionally been considered educationally meaningful [20]. A recent meta-analysis of Education intervention research found that effect sizes are rarely as large as .30 and hence that effect sizes of .50 would be considered very large indeed [21].

Table G. Estimates of Mean Change in the Scale-Up Study: Primary (grades 2/3) Per Protocol Sample

|  | **JUMP**  **Estimate (95%CL)**  **p-value** | **SB2**  **Estimate (95%CL)**  **p-value** | **JUMP minus SB2**  **Estimate (95%CL)**  **p-value** | **Effect Size** |
| --- | --- | --- | --- | --- |
| **Broad Math** | | | | |
| Year 1 | 1.2 (-0.1;2.5)  0.05 | 4.4 (3.0;5.8)  <.0001 | -3.2 (-4.9;-1.4)  **0.0004** | **-0.25** |
| Summer | -3.4 (-4.4;-2.4)  <.0001 | -4.7 (-5.9;-3.4)  <.0001 | 1.2 (-0.3;2.8)  0.1 | 0.13 |
| Year 2 | 0.7 (-0.4;1.9)  0.2 | -0.5 (-1.8;0.9)  0.5 | 1.2 (-0.4;2.8)  0.2 | 0.10 |
| **Broad Read** | | | | |
| Year 1 | 1.2 (0.6;1.8)  0.0003 | 0.9 (0.2;1.6)  0.02 | 0.3 (-0.6;1.2)  0.5 | 0.06 |
| Summer | -2.9 (-3.4;-2.4)  <.0001 | -2.5 (-3.1;-1.9)  <.0001 | -0.4 (-1.2;0.4)  0.3 | -0.12 |
| Year 2 | 0.5 (-0.1;1.2)  0.1 | -1.0 (-1.7;-0.2)  0.01 | 1.5 (0.6;2.4)  **0.001** | **0.25** |
| **Applied Problems** | | | | |
| Year 1 | 4.0 (2.7;5.2)  <.0001 | 6.0 (4.5;7.4)  <.0001 | -2.0 (-3.8;-0.2)  **0.03** | **-0.15** |
| Summer | -2.3 (-3.3;-1.3)  <0.0001 | -2.6 (-3.9;-1.3)  <.0001 | 0.3 (-1.4;2.0)  0.7 | 0.03 |
| Year 2 | 1.2 (-0.1;2.4)  0.07 | -0.3 (-1.8;1.1)  0.6 | 1.5 (-0.2;3.2)  0.09 | 0.12 |
| **Calculation** | | | | |
| Year 1 | -3.4 (-5.0;-1.8)  0.0001 | -0.4 (-2.3;1.4)  0.6 | -3.0 (-5.3;-0.6)  **0.01** | **-0.18** |
| Summer | -3.0 (-4.4;-1.5)  0.0001 | -5.7 (-7.5;-3.9)  <.0001 | 2.7 (0.4;5.1)  **0.02** | **0.17** |
| Year 2 | 0.0 (-1.7;1.6)  >0.9 | -0.2 (-2.1;1.7)  0.8 | 0.2 (-2.2;2.5)  0.9 | 0.01 |
| **Math Fluency** | | | | |
| Year 1 | -0.1 (-1.4;1.2)  0.8 | 0.3 (-1.1;1.8)  0.6 | -0.5 (-2.3;1.3)  0.6 | -0.04 |
| Summer | -3.9 (-5.0;-2.9)  <.0001 | -3.8 (-5.1;-2.5)  <.0001 | -0.1 (-1.9;1.6)  0.9 | -0.01 |
| Year 2 | 1.4 (0.1;2.7)  0.03 | 1.2 (-0.3;2.7)  0.1 | 0.2 (-1.7;2.0)  0.8 | 0.01 |
| **Problem-Solving Process** | | | | |
| Year 1 | 4.4 (2.8;5.9)  <.0001 | 5.4 (3.7;7.1)  <.0001 | -1.0 (-3.2;1.1)  0.3 | -0.13 |
| Year 2 | 6.2 (4.6;7.7)  <.0001 | 3.3 (1.6;5.1)  0.0003 | 2.8 (0.6;5.1)  **0.01** | **0.26** |
| **Curriculum Based Computation** | | | | |
| Year 1 | 7.2 (4.8;9.6)  <.0001 | 8.4 (5.7;11.1)  <.0001 | -1.2 (-4.6;2.1)  0.5 | -0.10 |
| Year 2 | 11.7 (9.1;14.2)  <.0001 | 13.6 (10.7;16.4)  <.0001 | -1.9 (-5.5;1.7)  0.3 | -0.11 |

Broad math comprises applied problems, calculation and math fluency and results for these measures and for broad reading are based on standard scores. Problem-solving process and curriculum based computation results are based on raw scores and percent correct, respectively. A positive effect size value indicates better performance in the JUMP group, a negative value better performance in the SB2 group. An effect size of .25 has traditionally been considered educationally meaningful [20]. A recent meta-analysis of Education intervention research found that effect sizes are rarely as large as .30 and hence that effect sizes of .50 would be considered very large indeed [21].

Table H. Estimates of Mean Change in the Scale-Up Study: Primary (grades 2/3) High Fidelity Sample

|  | **JUMP** | **SB2** | **JUMP minus SB2** | **\|Effect Size\|** |
| --- | --- | --- | --- | --- |
|  | **Estimate (95%CL)**  **p-value** | **Estimate (95%CL)**  **p-value** | **Estimate (95%CL)**  **p-value** |  |
| **Broad Math** | | | | |
| Year 1 | 0.9 (-0.7;2.5)  0.3 | 4.4 (2.4;6.3)  <.0001 | -3.5 (-5.7;-1.2)  **0.003** | **-0.24** |
| Summer | -3.2 (-4.4;-2.0)  <.0001 | -4.6 (-6.2;-2.9)  <.0001 | 1.4 (-0.7;3.4)  0.2 | 0.13 |
| Year 2 | 1.0 (-0.9;2.9)  0.3 | -2.5 (-4.8;-0.3)  0.03 | 3.5 (0.9;6.1)  **0.008** | **0.26** |
| **Broad Read** | | | | |
| Year 1 | 0.8 (-0.1;1.6)  0.07 | 0.6 (-0.4;1.7)  0.2 | 0.1 (-1.0;1.3)  0.8 | 0.03 |
| Summer | -3.0 (-3.6;-2.3)  <.0001 | -2.8 (-3.6;-1.9)  <.0001 | -0.2 (-1.2;0.9)  0.7 | -0.05 |
| Year 2 | 1.1 (-0.1;2.5)  0.07 | -1.0 (-2.3;0.4)  0.2 | 2.0 (.5;3.5)  **0.01** | **0.30** |
| **Applied Problems** | | | | |
| Year 1 | 3.4 (1.8;5.1)  0.0001 | 5.8 (3.7;8.0)  <.0001 | -2.4 (-4.8;0.0)  **0.05** | **-0.16** |
| Summer | -2.0 (-3.3;-0.7)  0.003 | -2.3 (-4.1;-0.4)  0.02 | 0.3 (-2.0;2.5)  0.8 | 0.02 |
| Year 2 | 1.3 (-0.8;3.4)  0.2 | -2.2 (-4.7;0.4)  0.09 | 3.5 (0.6;6.4)  **0.02** | **0.24** |
| **Calculation** | | | | |
| Year 1 | -3.2 (-5.2;-1.1)  0.003 | -1.6 (-4.2;0.9)  0.2 | -1.6 (-4.5;1.4)  0.3 | -0.09 |
| Summer | -2.8 (-4.6;-1.1)  0.002 | -5.2 (-7.5;-2.5)  <.0001 | 2.2 (-0.9;5.2)  0.2 | 0.13 |
| Year 2 | 0.1 (-2.4;2.7)  0.9 | -2.1 (-5.0;0.9)  0.2 | 2.2 (-1.2;5.6)  0.2 | 0.13 |
| **Math Fluency** | | | | |
| Year 1 | 0.3 (-1.2;1.9)  0.7 | 2.7 (0.7;4.6)  0.009 | -2.3 (-4.5;-0.1)  **0.04** | **-0.20** |
| Summer | -4.2 (-5.5;-2.9)  <.0001 | -5.1 (-6.9;-3.3)  <.0001 | 0.9 (-1.3;3.1)  0.4 | 0.09 |
| Year 2 | 1.6 (-0.6;3.8)  0.2 | 1.4 (-1.2;4.1)  0.3 | 0.1 (-2.9;3.2)  0.9 | 0.01 |
| **Problem-Solving Process** | | | | |
| Year 1 | 4.2 (2.4;6.0)  <.0001 | 5.2 (3.1;7.3)  <.0001 | -0.9 (-3.4;1.6)  0.4 | -0.12 |
| Year 2 | 7.6 (5.1;10.1)  <.0001 | 0.6 (-2.3;3.6)  0.7 | 7.0 (3.6;10.3)  **0.0001** | **0.54** |
| **Curriculum Based Computation** | | | | |
| Year 1 | 8.0 (4.9;11.1)  <.0001 | 7.9 (4.3;11.5)  <.0001 | 0.1 (-4.2;4.4)  >0.9 | 0.01 |
| Year 2 | 13.1 (8.8;17.5)  <.0001 | 16.9 (11.7;22.1)  <.0001 | -3.7 (-9.6;2.2)  0.2 | -0.16 |

Broad math comprises applied problems, calculation and math fluency and results for these measures and broad reading are based on standard scores. Problem-solving process and curriculum based computation results are based on raw scores and percent correct, respectively. A positive effect size value indicates better performance in the JUMP group, a negative value better performance in the SB2 group. An effect size of .25 has traditionally been considered educationally meaningful [20]. A recent meta-analysis of Education intervention research found that effect sizes are rarely as large as .30 and hence that effect sizes of .50 would be considered very large indeed [21].

**References**

1. OECD, Statistics Canada. Literacy for life: Further results from the adult literacy and life skills survey. Paris: OECD Publishing; 2011.
2. NCES. The nation's report card: A first look: mathematics and reading [Internet]. Washington D.C.: Institute of Education Sciences, U.S. Department of Education; 2013 p. 7. Available from: https://nces.ed.gov/nationsreportcard/subject/publications/main2013/pdf/2014451.pdf
3. ACT. The Condition of College and Career Readiness [Internet]. Iowa City: 2012 p. 4-5. Available from: https://www.act.org/content/dam/act/unsecured/documents/CCCR-2014-Iowa.pdf
4. Mighton J, Sabourin S, Klebanov A. JUMP Math. Toronto: JUMP Math;. 2008.
5. Solomon, T. & Mighton, J. Developing mathematical fluency: A strategy to help children learn their multiplication facts. Perspect Lang Lit. 2017; winter: 31-4.
6. Mighton, J. JUMP Math: multiplying potential. Not Am Math Soc, 2014;61:144-7.
7. Based on demographic data derived from a database of the results from a compulsory, provincial assessment in academic progress written annually at the end of grades 3 and 6, see [www.eqao.org](http://www.eqao.org). Data for the participating school board are based on the grade 6 students who participated in the assessment in the year preceding the study.
8. Following current recommendations by the CONSORT organization for randomized-controlled trials, we did not compare baseline characteristics of the two groups statistically.
9. Moher D, Hopewell S, Schulz KF, Montori V, Gøtzsche PC, Devereaux PJ, et al. CONSORT 2010 explanation and elaboration: Updated guidelines for reporting parallel group randomised trials. J Clin Epidemiol. 2010;63:e1–37.
10. Altman DG. Comparability of randomised groups. J R Stat Soc.1985;34:125–36.
11. Altman, Douglas G, Doré CJ. Randomisation and baseline comparisons. Lancet. 1990;335:149–53.
12. Kaufman AS, Kaufman NL. Kaufman Brief Intelligence Test, 2nd ed. Circle Pines: American Guidance Service; 2004.
13. Kaplan E, Fein D, Kramer D, Delis C, Morris R. WISC-III as a Process Instrument. San Antonio: Psychological Corporation; 1999.
14. Woodcock, RW, McGrew, KS, Mather N. Woodcock-Johnson III. Rolling Meadows: Riverside Publishing; 2001.
15. McGrew KS, Schrank FA, Woodcock RW. Technical Manual: Woodcock-Johnson III Normative Update. Rolling Meadows: Riverside Publishing; 2007.
16. Fuchs LS, Hamlett CL, Fuchs D. Monitoring Basic Skills Progress: Basic Math Computation. Austin: PRO-ED; 1998.
17. Wolf M. Denckla MB . Rapid Automatized Naming and Rapid Alternating Stimulus Tests. Austin: PRO-ED; 2005.
18. Ford L, Swart S, Negreiros J, Lacroix S, McGrew, KS. Use of the Woodcock-Johnson III NU Tests of Cognitive Abilities and Tests of Achievement with Canadian Populations (Woodcock Johnson III Assessment Service Bulletin No. 12) [Internet]. Rolling Meadows: Riverside Publishing;2010. Available from: <https://www.researchgate.net/publication/305391070_Woodcock-Johnson_Assessment_Bulletin_Number_12_Use_of_the_Woodcock-Johnson_III_NU_Tests_of_Cognitive_Abilities_and_Tests_of_Achievement_with_Canadian_Populations> [accessed Jun 04 2018].
19. Jaffe LE. Development, Interpretation, and Application of the *W* Score and the Relative Proficiency Index (Woodcock-Johnson III Assessment Service Bulletin No. 11) [Internet]. Rolling Meadows, IL: Riverside Publishing; 2009.
20. Tallmadge, GK. The Joint Dissemination Review Panel Idea Book. Washington, DC: National Institute of Education; 1977.
21. Lipsey MW, Puzio K, Yun C, Herbert MA, Steinka-Fry K, Cole MW. Translating the statistical representation of effects of education interventions into more readily interpretable forms [Internet]. Washington D.C: National Center for Special Education Research, Institute of Education Sciences, U.S. Department of Education; 2012 p. 33-37. Available from: https://ies.ed.gov/ncser/pubs/20133000/pdf/20133000.pdf
